# Manipulating Magnetism in Layered van der Waals Magnets via Structure–Property Engineering

**DOI:** 10.3390/nano16211362

**Published:** 2026-10-26

**Authors:** Xiangxiang Yu, Junkang Chen

**Affiliations:** School of Physics and Optoelectronic Engineering, Yangtze University, Jingzhou 434023, China

**Keywords:** van der Waals magnets, magnetic control, interlayer exchange, electric-field control, strain engineering

## Abstract

Selected van der Waals magnets retain magnetic order down to the monolayer limit. Their sensitivity to layer number, local bonding, carrier density, and interfaces provides routes to magnetic control for spintronic and quantum devices. In this review, we focus on the control of magnetism in representative two-dimensional systems and organize the field from the perspective of structure–property relations and matched control strategies. We show that weak interlayer exchange enables magnetic-field switching in few-layer magnets, that spin-layer locking supports electric-field control in bilayer CrI_3_, that doping-sensitive exchange allows carrier-density tuning in CrI_3_ and Fe_3_GeTe_2_, that stacking-dependent interlayer coupling makes pressure and twist effective structural knobs, that strong magnetoelastic coupling gives strain direct access to magnetic phase boundaries, and that interfacial exchange and proximity effects turn van der Waals heterostructures into active platforms for engineering emergent magnetic states. Representative material families are discussed in this context, including chromium trihalides, phosphorus chalcogenides, Ge- and Te-based layered magnets, transition-metal chalcogenides, MnBi_2_Te_4_-family compounds, and oxyhalides. Together, these studies show that the field is evolving from the discovery of intrinsic two-dimensional magnets toward the mechanism-guided design of controllable magnetic phases, magnetic heterostructures, and functional spintronic and quantum devices.

## 1. Introduction

Two-dimensional (2D) ferromagnetic materials have attracted broad interest because they combine magnetic order in atomically thin crystals with strong sensitivity to their structural and electronic environment [1,2]. Layered antiferromagnets extend this opportunity to compensated spin configurations and coupled electronic states. Their reduced dimensionality makes magnetic anisotropy, interlayer exchange, and interfaces central to the stability and control of magnetic order [3]. These interactions depend on local coordination, ligand bonding, and the arrangement of adjacent layers. Understanding these structural dependencies is therefore essential for selecting effective control methods. Beyond magnetism, nanostructure design, light modulation, and interface engineering also tune the performance of lithium-ion battery anodes [4,5,6].

Structural differences determine which magnetic interaction an external stimulus can tune. Chromium trihalides combine ligand-dependent anisotropy with stacking-sensitive interlayer exchange. Pressure and twist can therefore change the alignment of magnetic layers by altering their registry. In transition-metal phosphorus chalcogenides, sufficiently strong compression of bulk FePS_3_ and FePSe_3_ can instead drive a local spin crossover through changes in crystal-field splitting. In semiconducting Cr_2_Ge_2_Te_6_, deformation of Cr–Te bonds modifies ligand-mediated exchange, making strain a direct route to changing the magnetic transition temperature. The metallic Fe network in Fe_3_GeTe_2_ supports carrier control through changes in electronic filling, while interfaces introduce additional spin interactions and torques. In transition-metal dichalcogenides, coordination, stoichiometry, and lattice reconstruction influence the competition among magnetic and charge-ordered states. The MnBi_2_Te_4_ family provides another structural control route through the sequence of magnetic septuple layers and nonmagnetic quintuple layers. This sequence changes the separation and coupling of magnetic blocks, while film thickness affects magnetic compensation and topological transport. In layered oxyhalides such as VOCl, CrOCl, and FeOCl, distorted coordination creates directional exchange pathways and strong coupling between magnetic order and lattice symmetry. These features make pressure and strain particularly relevant to their magnetic response.

The effectiveness of a control method also depends on its microscopic action and the device geometry. A gate voltage can change carrier density, create a potential difference between layers, or deform a suspended magnetic membrane. These processes can modify electronic filling, the relative stability of layered magnetic configurations, or bond-mediated exchange. Thus, electrically induced magnetic responses require comparison in terms of the interaction being perturbed. Optical excitation can drive spin dynamics, while interfacial spin–orbit torques enable electrically written magnetic states [7,8]. Coupling magnetic order to topological bands or lattice polarization further supports quantum anomalous Hall transport and multistate information storage [9,10]. These applications motivate comparisons that connect a material’s structural features with the accessibility and limitations of each control route.

**Figure 1:**
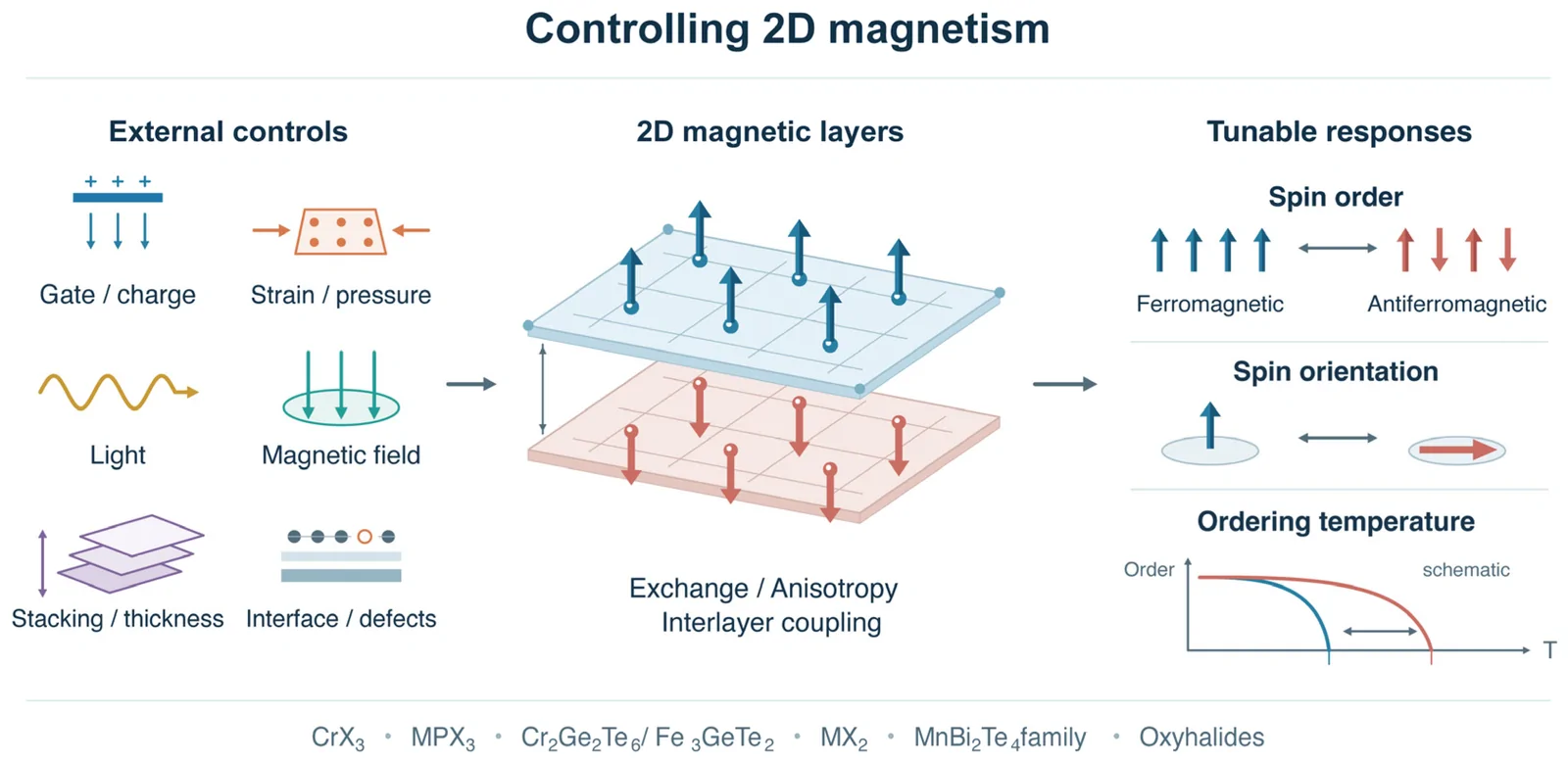
Schematic illustration of control approaches and tunable magnetic responses in six representative families of two-dimensional magnetic materials. The diagram highlights exchange interactions, magnetic anisotropy, and interlayer coupling, together with changes in magnetic order, spin orientation, and ordering temperature. Blue and red distinguish the illustrated magnetic layers, configurations, and spin orientations. The blue and red temperature-dependent curves illustrate lower and higher ordering temperatures, respectively. These curves are schematic and do not represent measurements on specific materials.

Existing reviews have clarified the fundamentals of 2D magnetism and surveyed magnetic materials and heterostructures. For example, Gibertini et al. emphasized the physical properties of CrI_3_ and Fe_3_GeTe_2_ and the opportunities offered by magnetic van der Waals heterostructures [3]. The present review complements these accounts by comparing how structural features govern the mechanisms, effectiveness, and limitations of magnetic control across six representative material families. It examines how the same stimulus acts on different structures and how different control methods modify the same magnetic interaction. This comparison connects local coordination, ligand bonding, stacking, and interfaces with the selection of magnetic-field, electrical, optical, and mechanical control strategies. Experimental observations are distinguished from theoretical predictions, and results from monolayers and few-layer devices are assessed separately from those obtained in bulk or chemically modified crystals. Figure 1 summarizes the main control approaches and the resulting magnetic responses across the six material families. Section 2 relates crystal structures to intrinsic magnetic mechanisms. Section 3 examines the corresponding control strategies, and Section 4 discusses shared challenges for reproducible magnetic and quantum devices.

## 2. Physics-Materials Correspondence in Representative Two-Dimensional Magnetic Materials

### 2.1. Chromium Trihalides and Anisotropic Superexchange in Ising- and XY-Type Two-Dimensional Ferromagnets

**Figure 2:**
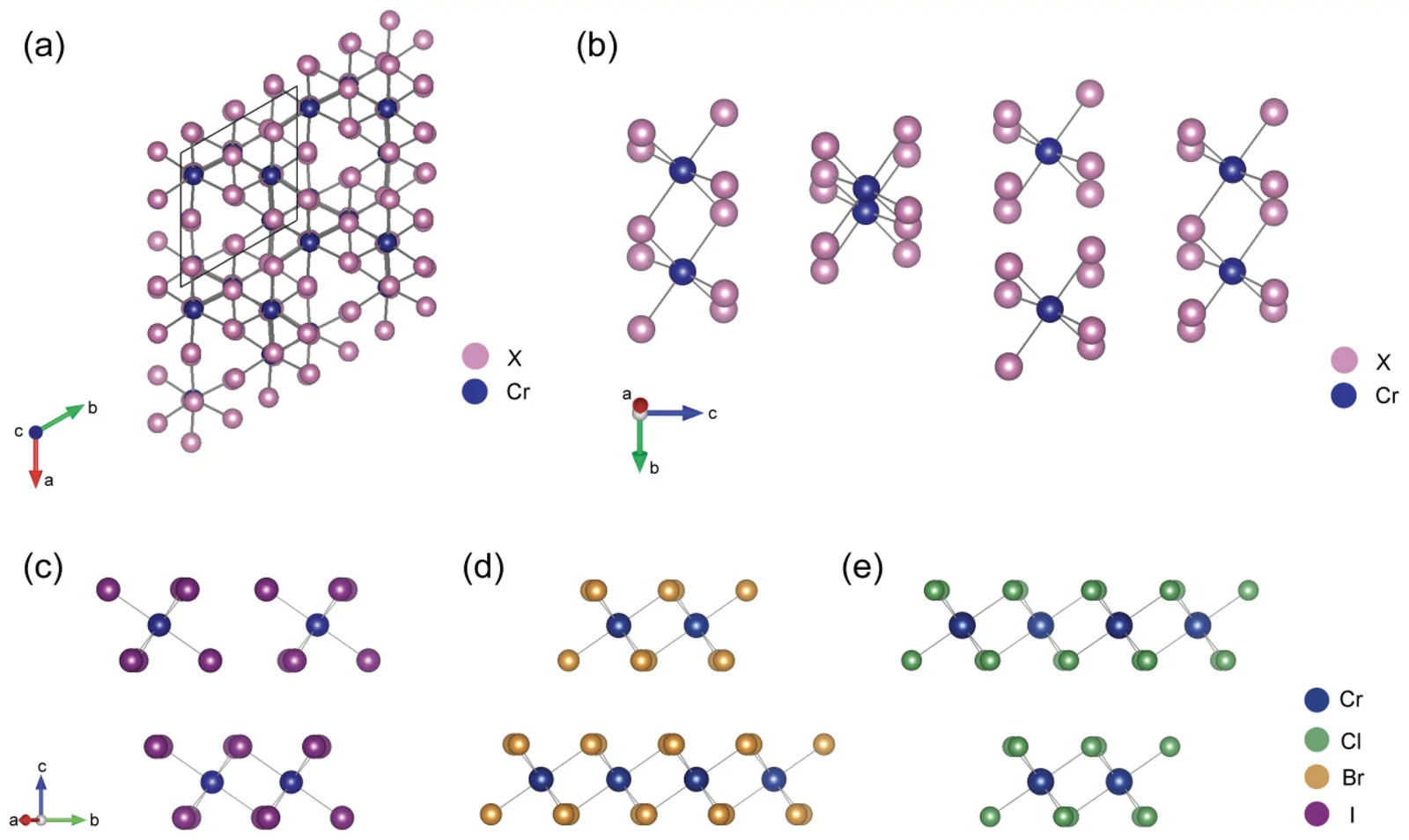
Crystal structures of chromium trihalides. (**a**) Top view of the honeycomb Cr sublattice. (**b**) Side view of the layered CrX_3_ lattice. (**c**–**e**) Side views of CrI_3_, CrBr_3_, and CrCl_3_, respectively. Cr and generic X sites are blue and pink; I, Br, and Cl are purple, orange, and green, respectively.

Chromium trihalides, CrX_3_ (X = Cl, Br, I), constitute a structurally coherent family of layered van der Waals magnets in which Cr^3+^ ions form a two-dimensional honeycomb lattice and are octahedrally coordinated by six halogen ligands, giving rise to layers of edge-sharing CrX_6_ octahedra weakly stacked along the c axis [11]. Figure 2 shows the honeycomb Cr sublattice and the layered structures of chromium trihalides. Across the series, CrCl_3_, CrBr_3_, and CrI_3_ preserve the same fundamental intralayer structure, including the honeycomb Cr network and the near-90° Cr-X-Cr bonding geometry, while the principal structural differences arise from the progressive increase in halogen size and polarizability from Cl to I. This ligand substitution systematically expands the lattice and interlayer spacing without altering the underlying crystallographic motif, so that the family is best viewed as a common octahedral honeycomb architecture continuously dilated from CrCl_3_ to CrBr_3_ and further to CrI_3_. In addition, CrCl_3_ and CrI_3_ exhibit temperature-dependent monoclinic and rhombohedral stacking polymorphs, indicating that the dominant crystallographic variation within chromium trihalides lies not in the local coordination environment of an individual layer but in the registry between adjacent layers [11]. Accordingly, CrCl_3_ may be regarded as the most compact member of the series, CrBr_3_ as an intermediate case, and CrI_3_ as the most expanded one, while all three remain variations in the same layered honeycomb-octahedral structural platform.

CrX_3_ is suitable for examining anisotropic superexchange because the three compounds preserve the same Cr honeycomb-octahedral structure but differ strongly in ligand size, covalency, and spin–orbit coupling. In each layer, the near-90° Cr-X-Cr geometry favors ferromagnetic superexchange between Cr^3+^ moments with a half-filled t_2g_ configuration. However, the halogen ligand controls the magnetic anisotropy. The weak spin–orbit coupling of Cl gives CrCl_3_ an easy-plane, XY-type character, whereas the stronger spin–orbit coupling of I gives CrI_3_ a more out-of-plane, Ising-like character. CrBr_3_ lies between these two limits [11]. This systematic evolution makes chromium trihalides a clean test system for understanding how ligand chemistry transforms a common ferromagnetic honeycomb layer from an XY-like magnet into an Ising-like magnet. At the same time, the weak interlayer exchange and stacking polymorphism produce different layered magnetic ground states in bulk and few-layer forms [1,12,13]. Thus, the intrinsic physics of CrX_3_ is governed by the interplay among local octahedral coordination, halogen-mediated superexchange, ligand-dependent magnetic anisotropy, and registry-sensitive interlayer coupling.

### 2.2. Anisotropy-Tunable Antiferromagnetism of Transition-Metal Phosphorus Chalcogenides Across the Ising-XXZ-XY Limits

**Figure 3:**
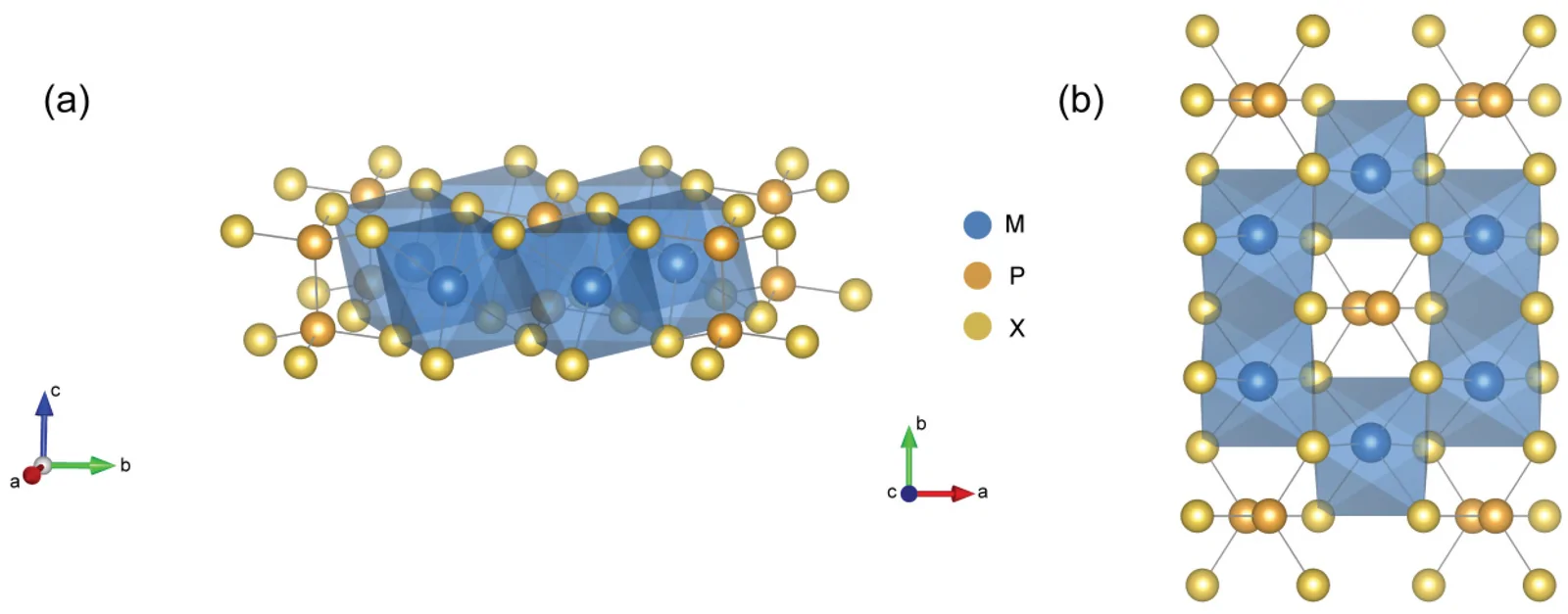
Crystal structure of transition-metal phosphorus chalcogenides. (**a**) Side view of an MPX_3_ layer. (**b**) Top view showing the honeycomb metal sublattice, edge-sharing MX_6_ octahedra, and P_2_X_6_ units. M, P, and X are blue, orange, and yellow, respectively. Blue polyhedra highlight MX_6_ coordination.

Transition-metal phosphorus chalcogenides, commonly represented by the formula MPX_3_ (M = transition metal; X = S, Se), crystallize as layered van der Waals compounds composed of atomically thin X-M/P-X sheets stacked along the out-of-plane direction. Figure 3 illustrates the layered MPX_3_ structure, the honeycomb metal sublattice, and the MX_6_ and P_2_X_6_ units. Within each layer, the transition-metal cations are typically octahedrally coordinated by six chalcogen atoms, forming a honeycomb-like network, while the phosphorus atoms occur as covalently bonded P_2_ dimers embedded within the chalcogen structure. The resulting structure can be viewed as a two-dimensional lattice of edge-sharing MX_6_ octahedra interconnected with (P_2_X_6_)^4−^ polyanionic units. Adjacent layers are held together by weak van der Waals interactions, which enable mechanical exfoliation down to the few-layer or monolayer limit [14]. This highly anisotropic crystal architecture provides the structural foundation for the pronounced low-dimensional magnetic, electronic, and lattice properties of the MPX_3_ family.

Against this structural background, MPX_3_ compounds are particularly suitable for examining anisotropy-controlled antiferromagnetism because the same layered honeycomb structure hosts markedly different spin Hamiltonians. The transition-metal ions provide localized d-electron moments, while the chalcogen ligands and (P_2_X_6_)^4−^ units mediate superexchange pathways that depend on bonding geometry, crystal-field splitting, and spin–orbit coupling. This intrinsic balance produces a natural sequence from Ising-type FePS_3_, to nearly Heisenberg-like MnPS_3_, and further to XXZ- or XY-like NiPS_3_ [15,16,17]. Their semiconducting or Mott-insulating character further stabilizes local moments and makes the coupling among exchange symmetry, magnetic anisotropy, and spin-lattice interactions especially clear [14]. Therefore, this section focuses on MPX_3_ as an intrinsic materials family for connecting crystal structure, electronic localization, and antiferromagnetic ground states, while external control strategies are discussed separately in the following section.

### 2.3. Ge- and Te-Based Layered Magnets from Superexchange-Driven Semiconductors to Itinerant Ising Ferromagnets

**Figure 4:**
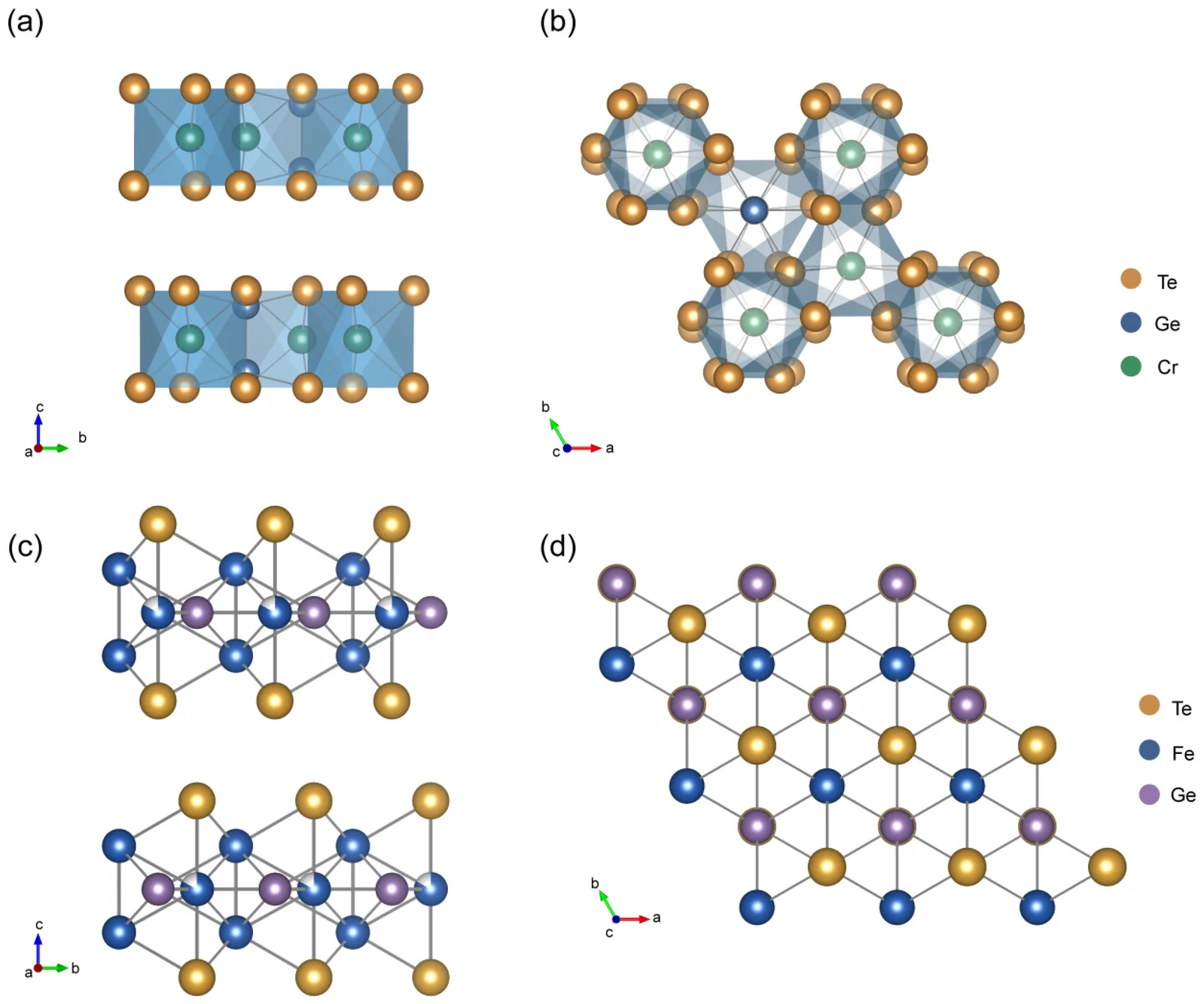
Crystal structures of Ge- and Te-based layered magnets. (**a**,**b**) Side and top views of Cr_2_Ge_2_Te_6_, showing CrTe_6_ octahedra and the honeycomb Cr sublattice. (**c**,**d**) Side and top views of Fe_3_GeTe_2_, showing Fe–Ge slabs bounded by Te layers. Cr, Ge, and Te in (**a**,**b**) are green, blue, and orange; Fe, Ge, and Te in (**c**,**d**) are blue, purple, and orange, respectively.

Cr_2_Ge_2_Te_6_ and Fe_3_GeTe_2_ represent two important yet structurally distinct classes of Ge- and Te-based layered magnets. Figure 4 compares the side and top views of Cr_2_Ge_2_Te_6_ and Fe_3_GeTe_2_. Cr_2_Ge_2_Te_6_ crystallizes in a rhombohedral van der Waals structure containing CrTe_6_ octahedra and Ge–Ge units bounded by Te, in which Cr ions are octahedrally coordinated by Te atoms and arranged into a honeycomb-like network within the layer. This structure gives rise to a relatively open and well-defined localized-spin lattice embedded in a Te-rich chalcogen environment [2,18]. In contrast, Fe_3_GeTe_2_ adopts a hexagonal layered structure composed of Fe-Ge slabs sandwiched between Te layers, where multiple Fe sites participate in a denser metallic network with stronger itinerant-electron character [19]. Thus, although both materials share the common feature of strong intralayer bonding combined with weak interlayer van der Waals coupling, their internal layer architectures are fundamentally different: Cr_2_Ge_2_Te_6_ is characterized by an octahedrally coordinated honeycomb-like magnetic structure, whereas Fe_3_GeTe_2_ exhibits a more compact and crystallographically complex Fe-based lattice. This structural contrast underlies the broader distinction between these two systems, namely, the more semiconducting and localized-spin nature of Cr_2_Ge_2_Te_6_ and the more metallic and itinerant-magnet character of Fe_3_GeTe_2_.

The intrinsic magnetic mechanisms of these two materials also differ sharply. In Cr_2_Ge_2_Te_6_, ferromagnetism is mainly associated with Te 5p-Cr e_g_ covalent hybridization and Cr–Te–Cr superexchange [2,18]. The near-90° Cr-Te-Cr geometry makes the magnetic ground state sensitive to local bonding, orbital hybridization, and spin-lattice coupling. This makes Cr_2_Ge_2_Te_6_ a useful test system for understanding how a semiconducting van der Waals lattice can host ferromagnetism through ligand-mediated exchange. Fe_3_GeTe_2_, by contrast, hosts correlation-assisted itinerant ferromagnetism from Fe-derived metallic bands near the Fermi level. Its strong perpendicular magnetic anisotropy stabilizes an Ising-like ferromagnetic state even in the atomically thin limit [19]. Therefore, Cr_2_Ge_2_Te_6_ and Fe_3_GeTe_2_ form a complementary pair. One captures a superexchange-driven semiconducting limit, while the other represents a metallic itinerant Ising-ferromagnetic limit. Their external controllability will be discussed in detail in the following section, where the focus shifts from intrinsic mechanisms to field-, carrier-, strain-, pressure-, and interface-driven magnetic manipulation.

### 2.4. Transition-Metal Chalcogenides with Covalent or Metallic Ferromagnetism and Competing Electronic Instabilities

**Figure 5:**
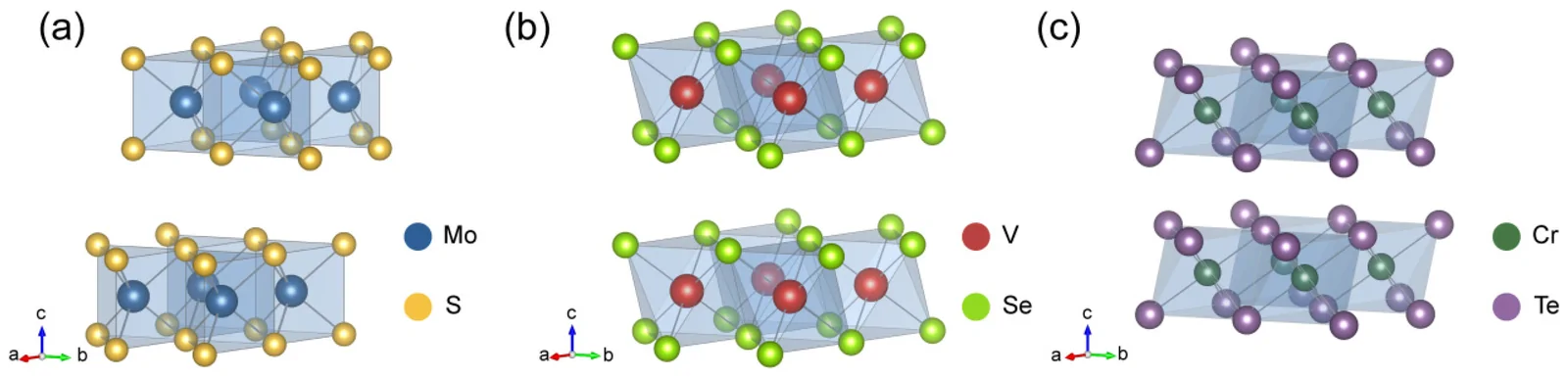
Coordination and stacking in transition-metal dichalcogenides. (**a**) Side view of 2H-MoS_2_ with trigonal-prismatic coordination. (**b**,**c**) Side views of 1T-VSe_2_ and 1T-CrTe_2_, respectively, with octahedral coordination. Polyhedra highlight the metal coordination in each X–M–X layer. Atom colors follow the panel legends.

Layered transition-metal chalcogenides with the MX_2_ stoichiometry are built from atomically thin X-M-X trilayers, where M denotes a transition metal and X denotes S, Se, or Te. In each trilayer, a plane of metal atoms is sandwiched between two chalcogen planes. Adjacent trilayers are connected mainly by weak van der Waals forces, which allows these compounds to be exfoliated or grown down to the monolayer limit. The metal atoms usually form a two-dimensional triangular lattice. Their local coordination can be trigonal prismatic in the 2H phase or octahedral in the 1T phase [20]. Figure 5 compares the trigonal-prismatic coordination in 2H-MoS_2_ with the octahedral coordination in 1T-VSe_2_ and 1T-CrTe_2_. Distorted 1T-derived structures and nonstoichiometric variants can also appear, especially in V-, Cr-, and Mn-based compounds [21,22,23]. This structural diversity changes the metal–chalcogen bond length, crystal-field splitting, d-orbital occupation, and interlayer coupling. As a result, MX_2_ chalcogenides provide a flexible structural platform for strain engineering, thickness control, defect regulation, charge doping, phase engineering, and interface design.

The magnetic behavior of MX_2_ chalcogenides originates from partially filled transition-metal d orbitals and their hybridization with chalcogen p states. In V-based compounds such as VS_2_, VSe_2_, and VTe_2_, narrow d bands often lie close to the Fermi level. This favors itinerant spin polarization, but it also makes the magnetic state compete strongly with charge-density-wave reconstruction and structural distortion. VSe_2_ is a typical case, because the proposed monolayer ferromagnetism lies close in energy to nonmagnetic, antiferromagnetic, and CDW-reconstructed states [22,24,25]. In Cr-based compounds such as CrS_2_, CrSe_2_, and CrTe_2_, magnetic order is mainly governed by the competition between direct Cr-Cr exchange and chalcogen-mediated Cr-X-Cr exchange. This competition can produce either ferromagnetic or antiferromagnetic ground states, depending on the lattice geometry and orbital filling [21]. For proposed Mn-based MX_2_ monolayers, calculations predict that strain and adsorbate-induced charge redistribution can alter magnetic order and anisotropy [23,26]. These predictions require experimental verification in samples with the specified structure and composition. By contrast, common Mo- and W-based MX_2_ materials are usually nonmagnetic in their pristine phases because their d-electron filling and band structures do not naturally favor spin polarization [20]. Therefore, ferromagnetism in MX_2_ chalcogenides is not a universal property. It appears only when d-electron filling, lattice symmetry, exchange interaction, and magnetic anisotropy jointly stabilize a spin-polarized state over competing nonmagnetic or charge-ordered phases.

### 2.5. MnBi_2_Te_4_ Family Materials with A-Type Antiferromagnetism Coupled to Topological Band Structure

**Figure 6:**
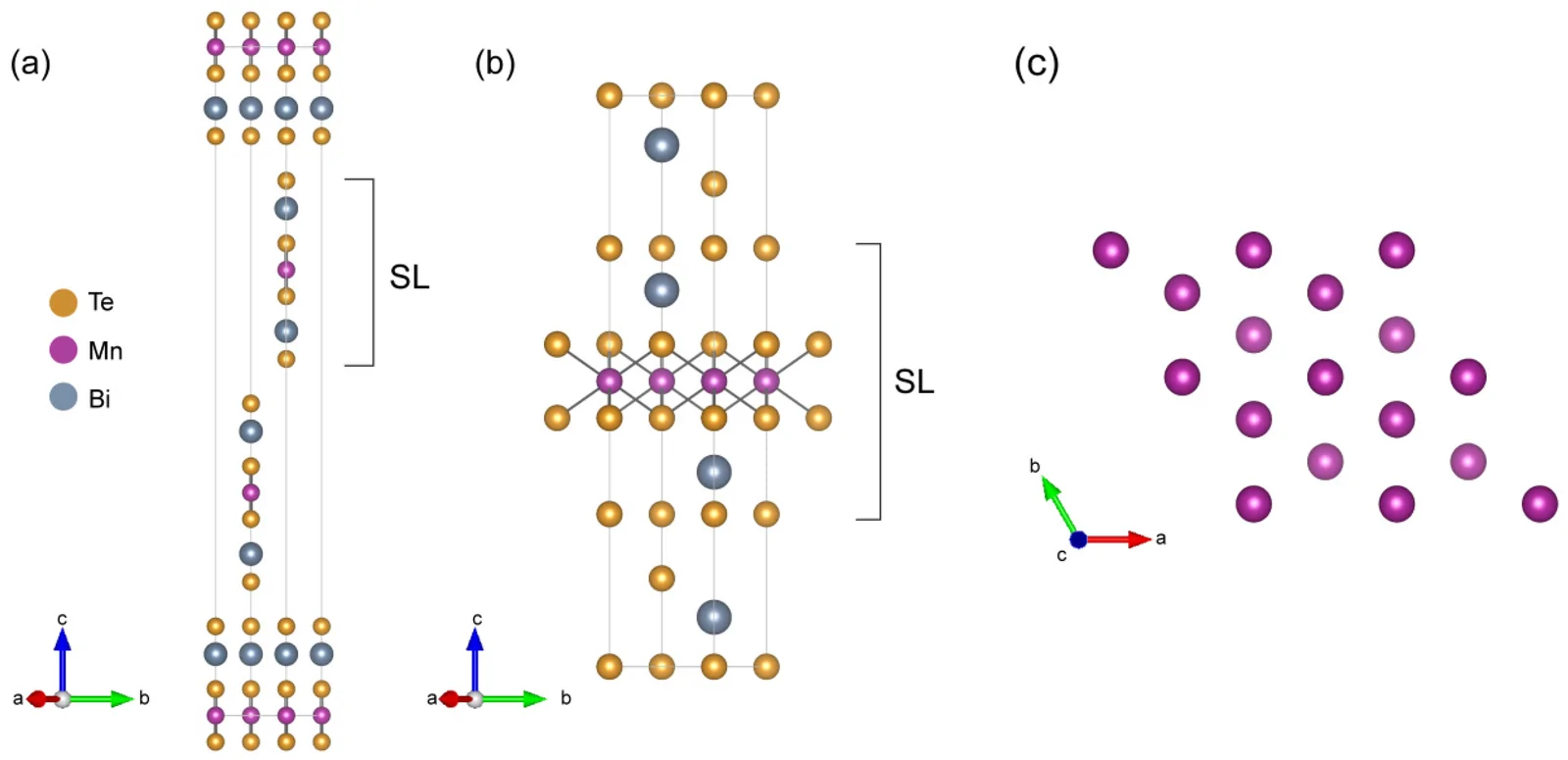
Layered structures of MnBi_2_Te_4_-family compounds. (**a**) MnBi_2_Te_4_ septuple layers (SLs) separated by van der Waals gaps. (**b**) MnBi_4_Te_7_ with magnetic SLs and nonmagnetic Bi_2_Te_3_ quintuple-layer blocks. (**c**) Top view of the triangular Mn sublattice. Brackets mark SLs. Mn, Bi, and Te are purple, blue-grey, and orange, respectively.

MnBi_2_Te_4_-family materials are tetradymite-derived van der Waals layered compounds built from close-packed triangular atomic sheets stacked along the c axis. The parent compound MnBi_2_Te_4_ crystallizes in the rhombohedral R$\bar{3}$m (No. 166) structure and consists of Te-Bi-Te-Mn-Te-Bi-Te septuple layers, which can be viewed as Bi_2_Te_3_-type quintuple layers with an inserted Mn-Te bilayer at the center; these septuple blocks are separated by van der Waals gaps, giving the material a strongly anisotropic quasi-two-dimensional structure [27]. In the broader family, the homologous series is commonly expressed as (MnBi_2_Te_4_)(Bi_2_Te_3_)_m_, where nonmagnetic Bi_2_Te_3_ quintuple layers are inserted between neighboring MnBi_2_Te_4_ septuple layers, generating compounds such as MnBi_4_Te_7_, MnBi_6_Te_10_, MnBi_8_Te_13_, MnBi_10_Te_16_, MnBi_12_Te_19_, and MnBi_14_Te_22_. This modular septuple–quintuple stacking preserves the same basic tetradymite motif while systematically increasing the separation between magnetic building blocks, thereby providing a structurally tunable platform for controlling interlayer coupling and related physical properties across the series [28]. Figure 6 illustrates the septuple-layer and quintuple-layer stacking and the triangular Mn sublattice in representative MnBi_2_Te_4_-family compounds.

The intrinsic physics of MnBi_2_Te_4_-family materials comes from the coupling between Mn-derived magnetism and Bi/Te-derived topological bands. The Bi and Te p orbitals, together with strong spin–orbit coupling, provide the inverted band structure inherited from tetradymite topological insulators. The Mn layers introduce localized magnetic moments into this topological structure. Within each septuple layer, Mn moments are ferromagnetically aligned, while adjacent septuple layers are coupled antiferromagnetically along the out-of-plane direction. This produces an A-type antiferromagnetic ground state in the parent compound [27]. The coexistence of out-of-plane magnetic order, strong spin–orbit coupling, and van der Waals layering makes these materials especially suitable for testing how intrinsic antiferromagnetism modifies topological surface states and bulk band topology. In this sense, MnBi_2_Te_4_-family compounds are not only layered magnetic crystals, but also intrinsic antiferromagnetic topological systems in which magnetic order and electronic topology are built into the same crystallographic structure.

### 2.6. Oxyhalides as Low-Symmetry Anisotropic Antiferromagnets with Strong Magnetoelastic Coupling

**Figure 7:**
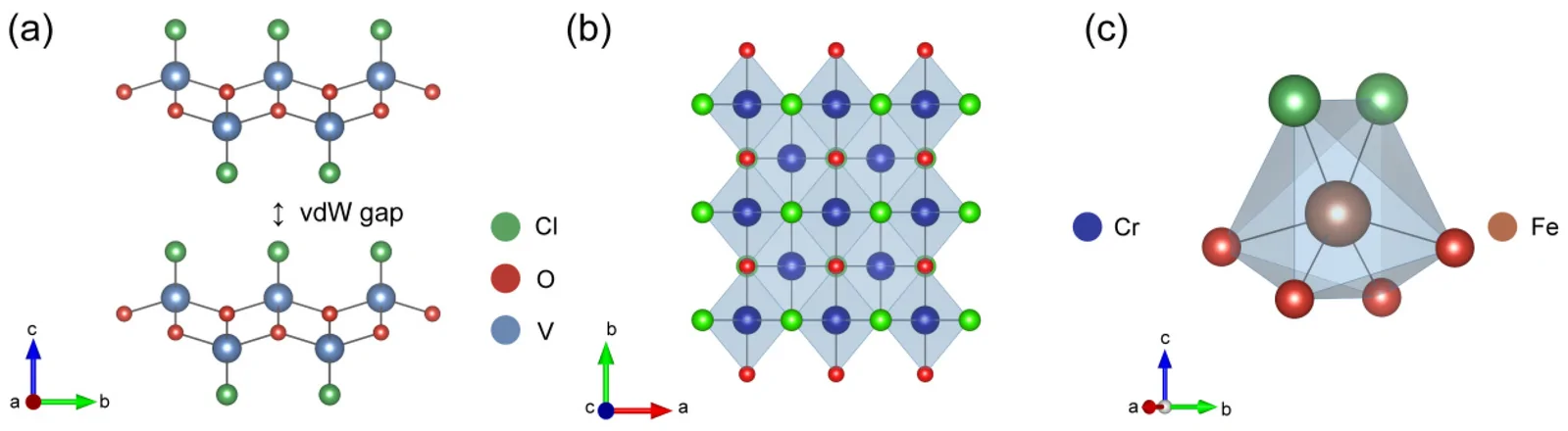
Crystal structures and coordination in layered oxyhalides. (**a**) VOCl slabs separated by a Cl–Cl van der Waals gap. (**b**) Top view of the distorted CrO_4_Cl_2_ network in CrOCl. (**c**) Local FeO_4_Cl_2_ coordination. V, Cr, Fe, O, and Cl are blue-grey, dark blue, brown, red, and green, respectively. Polyhedra highlight metal coordination.

VOCl, CrOCl, and FeOCl belong to the FeOCl-type layered family and crystallize in the orthorhombic space group Pmmn under ambient conditions. Their structures consist of distorted MO_4_Cl_2_ octahedra (M = V, Cr, or Fe) connected through shared edges and corners. These units form double layers in the ab plane, while adjacent slabs are separated along the c axis by van der Waals gaps between Cl-terminated surfaces [29,30,31]. Figure 7 shows the layered structures and distorted metal coordination in VOCl, CrOCl, and FeOCl. This arrangement combines weak interlayer coupling with pronounced structural anisotropy within each layer.

Diffraction studies of bulk VOCl, CrOCl, and FeOCl show that antiferromagnetic ordering is accompanied by a distortion from orthorhombic to monoclinic symmetry [29,30,31]. This coupling between magnetic order and lattice deformation modifies competing exchange pathways and can relieve magnetic frustration. In VOCl, the strong magnetoelastic response reflects the sensitivity of exchange interactions to V–O–V bond angles and V–V distances [29]. CrOCl develops stripy antiferromagnetic order with pronounced in-plane anisotropy [10,30]. FeOCl exhibits an incommensurate antiferromagnetic modulation coupled to the lattice distortion [31].

## 3. Structure–Property Relations and Matched Control Strategies in Two-Dimensional Magnets

The following sections compare how specific structural features determine the effectiveness of magnetic control. Layer registry changes interlayer exchange, bond deformation modifies exchange geometry, and carrier redistribution alters the relative stability of magnetic states. Sample thickness and chemical modification are specified separately because monolayers, few-layer devices, bulk crystals, and intercalated compounds provide different evidence for low-dimensional magnetism.

### 3.1. Spin-Dependent Transport, Stacking, and Voltage Control in Chromium Trihalides

**Figure 8:**
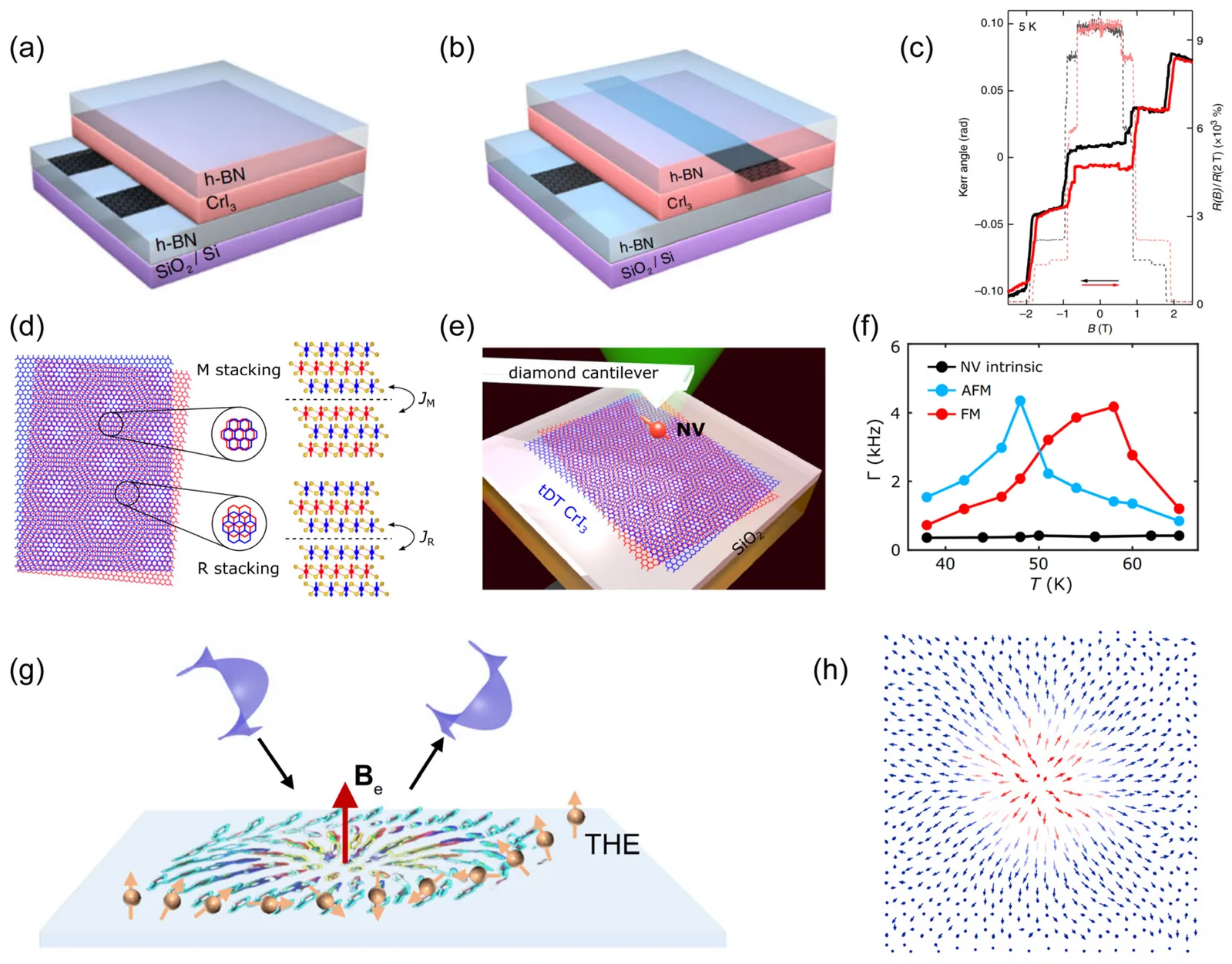
Structure-guided control in CrI_3_. (**a**,**b**) Lateral and vertical device schematics. (**c**) Kerr rotation and magnetoresistance measured on the same device at 5 K. Solid curves show the Kerr angle, and dashed curves show the resistance ratio. Red and black denote increasing and decreasing magnetic-field sweeps, respectively. From Wang et al. [34], figures 1a,c and 5a. (**d**) Moiré stacking model showing M- and R-stacked regions in twisted double-trilayer CrI_3_. Blue and red mark Cr sites in the upper and lower layers adjacent to the twisted interface, respectively. Yellow spheres represent I atoms, and the colored arrows indicate local Cr magnetic moments. (**e**) Scanning nitrogen-vacancy (NV) magnetometry geometry. The blue and red lattice colors follow the layer assignments in (**d**), and the sphere at the cantilever tip represents the NV sensor. (**f**) Temperature-dependent NV spin-relaxation rates above antiferromagnetic and ferromagnetic regions, with intrinsic NV relaxation shown for comparison. From Li et al. [35], figures 1a, 2a and 3c. (**g**) Schematic of the topological Hall effect and topological magnetic circular dichroism induced by an emergent magnetic field. From Wu et al. [37], figure 1a. (**h**) AI-assisted schematic redrawing of a skyrmion-like spin texture based on the inset of figure 3d in the same work. Arrows illustrate local spin directions. Red and blue distinguish illustrative regions of the texture without quantitative color calibration; neither the arrows nor the colors reproduce the original numerical spin configuration. Source materials © The Author(s) 2018, 2024, and 2026, respectively, under CC BY 4.0 (https://creativecommons.org/licenses/by/4.0/). Panels cropped, rearranged, and relabeled.

Chromium trihalides separate two structural contributions to magnetic control. The halogen environment governs intralayer superexchange and magnetic anisotropy, while the registry between adjacent layers controls weak interlayer exchange [11,12,13]. This distinction explains why pressure and twisting can change the alignment of magnetic layers without changing their chemical composition. Gate control introduces a different perturbation through carrier density and the electrostatic environment [32,33]. Figure 8 connects these mechanisms with tunneling readout, spatially varying magnetic order, and voltage-dependent spin textures.

The lateral and vertical devices in Figure 8a,b illustrate why current direction matters in a layered magnetic semiconductor. In the vertical geometry, carriers traverse the CrI_3_ barrier and therefore probe its magnetic configuration through tunneling. Wang et al. compared Kerr rotation and magnetoresistance on the same device at 5 K. The corresponding steps occur at matching magnetic fields, as shown in Figure 8c [34]. This agreement connects resistance changes to magnetic transitions and provides a basis for spin-dependent readout. It does not, by itself, uniquely resolve the microscopic spin arrangement of every intermediate resistance state. Encapsulation and contact geometry are integral parts of the demonstrated device.

Twisting converts stacking-dependent exchange into a spatially varying interaction. In small-angle twisted double-trilayer CrI_3_, different local registries favor ferromagnetic or antiferromagnetic interlayer alignment. Figure 8d illustrates the M- and R-stacked regions, and Figure 8e shows the scanning nitrogen-vacancy magnetometry geometry. Li et al. measured temperature-dependent spin fluctuations above the two types of magnetic regions [35]. The relaxation maxima in Figure 8f indicate transition temperatures separated by approximately 10 K. Their mean-field analysis connects this difference to stacking-dependent interlayer exchange. Thus, local registry controls both the preferred spin alignment and its thermal stability.

Magnetic textures can also extend beyond the structural moiré period. Wong et al. used scanning nitrogen-vacancy microscopy to study twisted double-bilayer CrI_3_ and observed magnetic textures with characteristic sizes of about 300 nm near a twist angle of 1.1° [36]. The magnetic length scale exceeded the underlying moiré wavelength. Stray-field maps and atomistic simulations were consistent with antiferromagnetic Néel-type skyrmions. The simulations linked their formation to competing exchange interactions, magnetic anisotropy, and interfacial Dzyaloshinskii–Moriya interactions. This comparison shows that twist engineering requires consideration of both local stacking and magnetic interactions extending across multiple moiré cells. Lattice relaxation and local disorder remain uncertainties in the microscopic interpretation.

Electrical control can also alter intralayer spin textures. Wu et al. used reflective magnetic circular dichroism (RMCD) to investigate the gate-dependent response of monolayer CrI_3_ at 10 K [37]. Figure 8g illustrates how the emergent magnetic field associated with a topological spin texture can contribute to electrical and optical Hall responses. The authors combined the measured RMCD response with spin-lattice simulations to investigate the formation of voltage-controlled topological spin textures. Figure 8h provides an illustrative redrawing of a skyrmion-like spin configuration. The interpretation rests on the combined magneto-optical measurements and modeling, rather than direct real-space imaging of the texture.

These examples complement established field, pressure, and carrier control. Pressure can strengthen interlayer coupling and change stacking, whereas a layer potential difference and charge doping influence different terms in the bilayer magnetic energy [32,33,38]. In CrCl_3_, thickness-dependent polarization fields and antiferromagnetic resonance instead emphasize easy-plane anisotropy and weak interlayer exchange [12,39]. Helicity-dependent optical writing in CrI_3_ extends control into the time domain [40].

### 3.2. Dimensionality, Spin Crossover, and Interfacial Exchange in MPX_3_

In MPX_3_ compounds, the metal ion determines the local electronic configuration, while the surrounding ligands control exchange pathways and magnetic anisotropy [14,15,16,17]. Thickness reduction weakens interlayer coupling and increases the importance of fluctuations and residual in-plane anisotropy. Compression can produce a more substantial change by modifying the crystal-field splitting and the local spin state. At a magnetic interface, reducing the layer separation can strengthen exchange across the van der Waals gap. These mechanisms act on different parts of the magnetic energy and should be assessed through the corresponding ordering, spin-state, or exchange-bias measurements.

**Figure 9:**
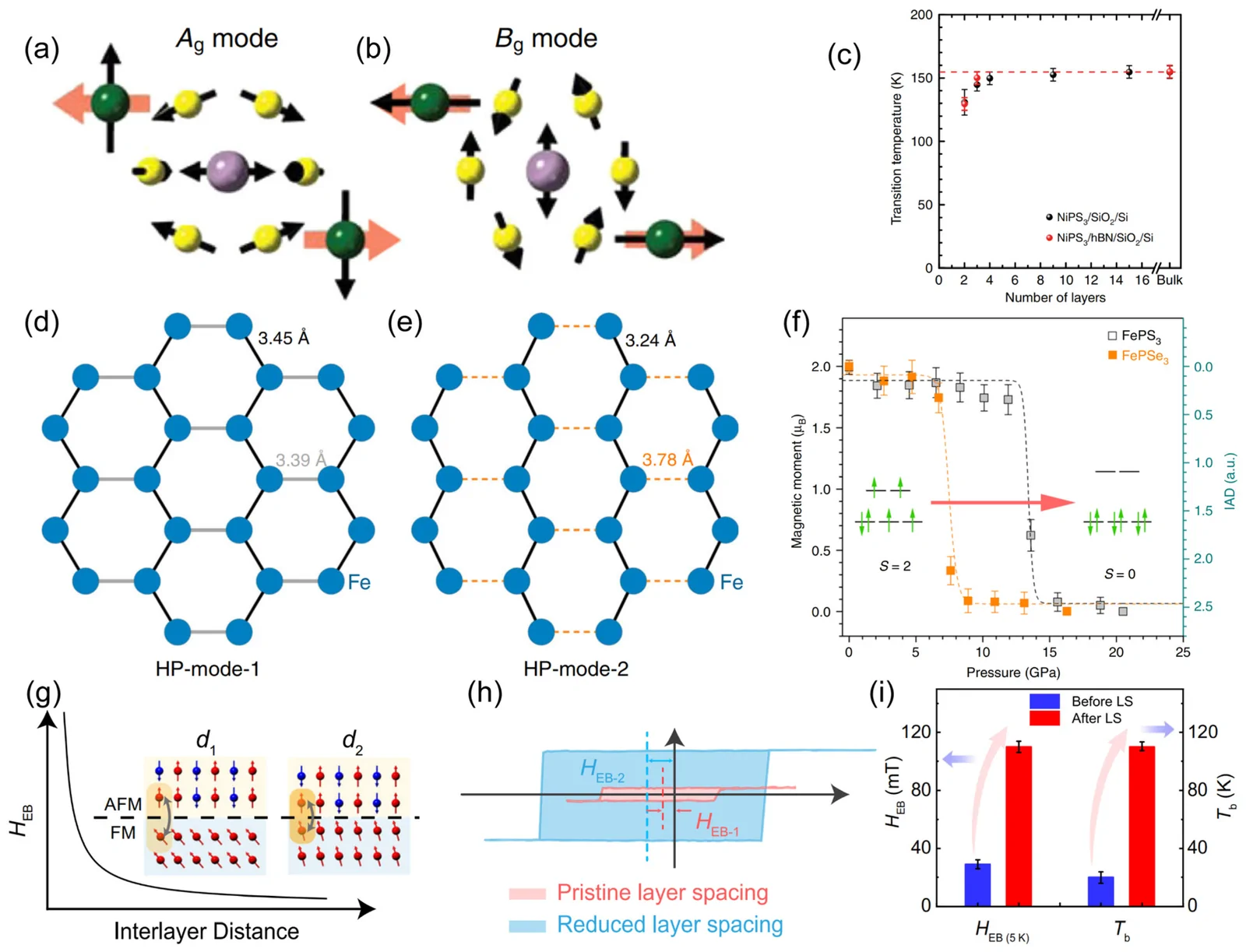
Dimensionality, pressure, and interface control in MPX_3_. (**a**,**b**) Atomic-displacement patterns of the (A_g_) and (B_g_) phonon modes in NiPS_3_. Green, purple, and yellow spheres represent Ni, P, and S atoms, respectively. Black arrows indicate atomic displacements, and red arrows indicate Ni spin orientations. (**c**) Raman-derived antiferromagnetic transition temperatures as a function of layer number. Black and red symbols correspond to samples on SiO_2_/Si and h-BN/SiO_2_/Si substrates, respectively. The red dashed line marks the bulk Néel temperature of 155 K. Panels (**a**,**b**) and (**c**) are from Kim et al. [17], figures 4d and 5b, respectively. (**d**,**e**) Proposed high-pressure FePSe_3_ honeycomb structures, denoted HP-mode-1 and HP-mode-2. (**f**) Pressure-dependent Fe magnetic moment and integrated absolute difference (IAD) derived from X-ray emission spectra, indicating a high-spin to low-spin crossover in FePS_3_ and FePSe_3_. From Wang et al. [42], figures 2c and 5a,b. (**g**) Schematic dependence of exchange bias on antiferromagnet–ferromagnet interlayer separation. Pale yellow and blue regions represent the antiferromagnetic and ferromagnetic layers, respectively. Red and blue spin symbols distinguish opposite spin orientations, and curved arrows indicate interfacial exchange coupling. (**h**) Schematic hysteresis loops illustrating enhanced exchange bias at reduced layer spacing. (**i**) Exchange-bias field and blocking temperature of FePSe_3_/Fe_3_GeTe_2_ before and after laser shocking (LS). Panels (**g**,**h**) and (**i**) are from Huang et al. [43], figures 1a and 2e, respectively. Panels (**a**–**c**), (**d**–**f**), and (**g**–**i**) © The Author(s) 2019, 2018, and 2023, respectively, under CC BY 4.0 (https://creativecommons.org/licenses/by/4.0/). Panels cropped, rearranged, and relabeled.

NiPS_3_ illustrates why the inferred dimensional limit of magnetic order depends on both the measurement and the assumed anisotropy. The A_g_ and B_g_ displacement patterns in Figure 9a,b show the phonons used to probe spin–lattice coupling. Kim et al. tracked magnetic Raman features and phonon-frequency differences in exfoliated samples on SiO_2_/Si and h-BN/SiO_2_/Si substrates [17]. The few-layer transition temperatures are summarized in Figure 9c. In monolayers, the characteristic signatures of bulk-like ordering were strongly suppressed, with magnetic fluctuations persisting to the lowest measured temperatures. Similar results on the two substrates indicated that this observation could not be assigned simply to the choice between these supports.

Cheon et al. subsequently investigated monolayer NiPS_3_ in fully h-BN-encapsulated field-effect devices with graphene contacts [41]. Conductance and magnetoresistance measurements resolved two magnetic transitions and supported a low-temperature ordered state governed by weak in-plane hexagonal anisotropy. Their interpretation differs from an ideal easy-plane model with continuous spin symmetry because a discrete anisotropy can alter the low-temperature phase. The two studies also probe different observables, including spin-dependent lattice vibrations and electrical transport. The absence of a bulk-like Raman signature therefore does not exclude every form of monolayer magnetic order. Probe sensitivity, temperature range, and residual anisotropy provide possible explanations for the contrasting conclusions, but their relative contributions require measurements under matched sample conditions.

High-pressure measurements on bulk FePS_3_ and FePSe_3_ crystals probe changes in the Fe electronic state and should be distinguished from measurements on isolated monolayers. Figure 9d,e presents two proposed high-pressure structures of FePSe_3_, with different degrees of distortion in the Fe honeycomb lattice. X-ray emission analysis reveals a pronounced reduction in the Fe moment under compression in FePS_3_ and FePSe_3_, consistent with a crossover from a high-spin state to a low-spin state, as shown in Figure 9f [42]. Structural and transport measurements in the same study connect this change with lattice collapse and metallization. Superconductivity was additionally reported in compressed FePSe_3_. Figure 9f tracks the spin-state evolution rather than superconductivity, while Figure 9d,e shows proposed structural models rather than successive stages of a confirmed transition.

Interlayer spacing provides a direct interface-design variable in FePSe_3_/Fe_3_GeTe_2_. Huang et al. used laser shocking to reduce the van der Waals separation and strengthen exchange bias [43]. Figure 9g illustrates the expected enhancement of interfacial exchange as the antiferromagnetic and ferromagnetic layers approach each other. The schematic hysteresis loops in Figure 9h show the corresponding increase in exchange-bias magnitude. The experimental comparison in Figure 9i records an exchange-bias increase from about 29 to 111 mT and a blocking-temperature increase from 20 to 110 K. These results support stronger interfacial coupling after laser-shock processing. The increased blocking temperature characterizes the persistence of exchange bias in the heterojunction and should not be equated with a change in the ordering temperature of isolated FePSe_3_.

Other control routes act on complementary terms in the same materials. Magnetoelectric cooling can select MnPS_3_ domain populations, while resonant optical excitation can drive coherent spin dynamics in NiPS_3_ [7,44]. Ligand substitution changes bonding and anisotropy, with different ordering-temperature trends for Mn- and Ni-based compounds [45]. Molecular intercalation and electron doping can further alter the magnetic state, but structural expansion and carrier injection are coupled in these experiments [46,47].

### 3.3. Magnetoelastic Control and Functional Interfaces in Ge- and Te-Based Magnets

Cr_2_Ge_2_Te_6_ and Fe_3_GeTe_2_ respond to control through different electronic structures. In semiconducting Cr_2_Ge_2_Te_6_, changes in Cr–Te bonding modify ligand-mediated exchange, which makes elastic deformation a direct route to shifting the magnetic transition [2,18,48]. In metallic Fe_3_GeTe_2_, Fe-derived states near the Fermi level and strong perpendicular anisotropy support carrier and interface control [19]. Interfaces can additionally introduce antisymmetric exchange or inject spin angular momentum [8,49]. The relevant comparison is therefore between strain-induced changes in exchange geometry and interface-induced changes in spin interactions or torque.

**Figure 10:**
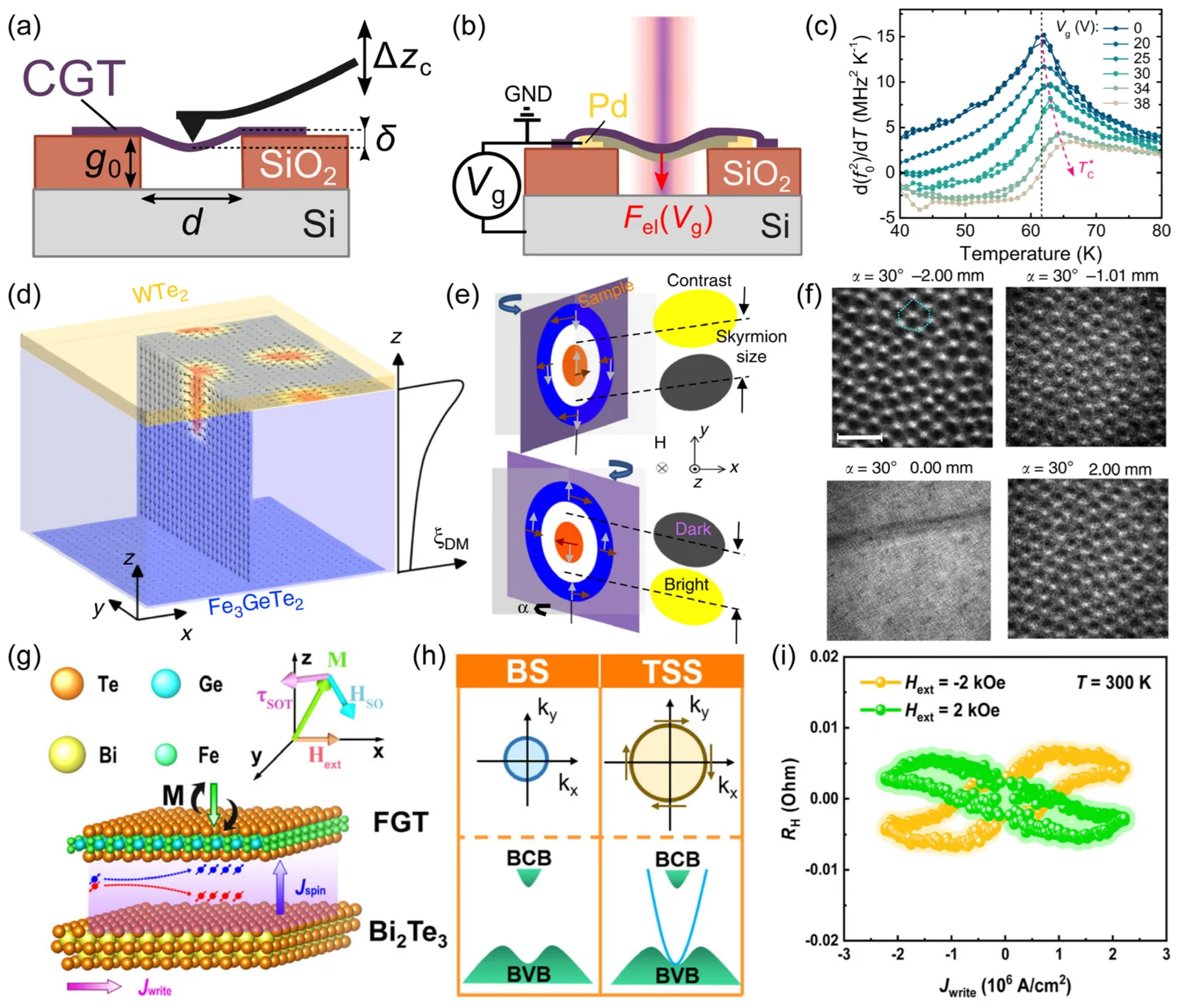
Mechanical and interfacial control of Ge- and Te-based magnets. (**a**) Indentation geometry for a suspended Cr2Ge2Te6 membrane. (**b**) Gated-membrane resonator showing electrostatic deflection. (**c**) Temperature derivatives of the squared resonance frequency under different gate voltages. From Šiškins et al. [48], figures 1b and 5c,f. (**d**) Simulated three-dimensional skyrmion texture in WTe_2_/Fe_3_GeTe_2_ with a depth-dependent interfacial Dzyaloshinskii–Moriya interaction. Yellow and pale blue identify the WTe_2_ and Fe_3_GeTe_2_ slabs, respectively. Arrows show local spin directions, and warm-colored regions highlight the skyrmion cores against the surrounding magnetic background. (**e**) Schematic of Néel-skyrmion contrast in tilted Lorentz transmission electron microscopy. Orange and blue disks encode magnetization along +z and −z, respectively. Brown arrows show the in-plane magnetization component, gray arrows indicate the Lorentz force on the electron beam, and curved blue arrows indicate sample tilting. Yellow and dark disks represent bright and dark image contrast. (**f**) Experimental defocus series for WTe_2_/40-layer Fe_3_GeTe_2_ at 180 K and 510 Oe with a sample tilt of 30°. Scale bar, 500 nm. From Wu et al. [49], figure 3d, three-dimensional model, and figures 3a,b. (**g**) Spin-current injection and spin–orbit torque in Bi_2_Te_3_/Fe_3_GeTe_2_. (**h**) Schematics of bulk states (BS) and topological surface states (TSS). (**i**) Current-induced switching at 300 K under assisting in-plane fields of ±2 kOe. From Wang et al. [8], figures 2a and 5a,c. Panels (**a**–**c**), (**d**–**f**), and (**g**–**i**) © The Author(s) 2022, 2020, and 2023, respectively, under CC BY 4.0 (https://creativecommons.org/licenses/by/4.0/). Panels cropped, rearranged, and relabeled.

Suspending Cr_2_Ge_2_Te_6_ makes its mechanical deformation both controllable and measurable. The indentation and gated-resonator schematics in Figure 10a,b show how elastic characterization and electrostatic deflection can be implemented in thin membranes. Šiškins et al. tracked magnetoelastic anomalies in the resonance frequency of suspended heterostructures [48]. The temperature derivatives of the squared resonance frequency in Figure 10c show a shift in the transition-related peak as the gate bias changes the membrane strain. The study reports a Curie-temperature enhancement of 2.5 ± 0.6 K for an applied strain of 0.026%. Here the voltage acts through electrostatic force and membrane deformation, so the response should not be assigned to pure carrier-density control. Resonator geometry and the nonmagnetic supporting layer influence the strain transmitted to the magnet.

An interface can favor spatially nonuniform spin order. In WTe_2_/Fe_3_GeTe_2_, broken interfacial symmetry and spin–orbit coupling support a Dzyaloshinskii–Moriya interaction and Néel-type skyrmions [49]. Figure 10d shows a simulated three-dimensional spin texture for an interaction that decays away from the interface. Figure 10e illustrates how sample tilt produces magnetic contrast in Lorentz transmission electron microscopy. The experimental defocus series in Figure 10f was obtained at 180 K and 510 Oe with a sample tilt of 30°. The appearance and reversal of the image contrast with defocus connect the magnetic texture to the imaging response. Interpreting these images requires both the spin model and the measurement geometry.

The Bi_2_Te_3_/Fe_3_GeTe_2_ interface provides a route to electrical writing. The schematic in Figure 10g identifies the charge-current path, injected spin current, and resulting torque on the ferromagnet. The band sketches in Figure 10h distinguish bulk states from topological surface states. Wang et al. demonstrated current-induced switching at 300 K under an assisting in-plane field of ±2 kOe, as shown in Figure 10i [8]. The reported switching current density is approximately 2.2 × 10^6^ A cm^−2^. These results establish room-temperature operation in the studied heterostructure, with an assisting field required by the demonstrated protocol. Comparisons of switching efficiency across devices also require consideration of magnetic thickness, current partition between layers, and the switching protocol.

Mechanical strain, chemical modification, and electronic gating modify exchange and anisotropy through different mechanisms. Organic-ion intercalation in bulk Cr_2_Ge_2_Te_6_ crystals couples electron transfer to layer expansion [18]. Pressure measurements on bulk samples probe changes in spin–lattice coupling and magnetic anisotropy [50,51]. These results identify control mechanisms in chemically modified or compressed crystals, whose responses require separate validation in isolated atomic layers. In Fe_3_GeTe_2_-based systems, ionic gating, ferroelectric interfaces, and proton-mediated changes in interlayer coupling provide further control channels [52,53,54].

Related Fe-based tellurides extend this comparison to composition and atomic-site occupancy. Fe_3_GaTe_2_ films grown by molecular-beam epitaxy retain ferromagnetism above room temperature on a wafer scale [55]. Separate studies demonstrate room-temperature spin–orbit-torque switching [56], nonvolatile modulation through ferroelectric interfaces [57], and room-temperature skyrmions [58]. These responses show how an itinerant magnetic layer can support different functions through its interfaces and magnetic texture. Their comparison must retain the individual film thicknesses and device conditions because the reported capabilities were obtained in different samples.

Atomic-site order provides another control variable. In Fe_5−δ_GeTe_2_ single crystals, thermal treatment changes Fe-vacancy ordering and produces reversible switching between persistent structural and electronic states [59]. This result links the arrangement of partially occupied Fe sites to the electronic structure of the magnetic host. It establishes a route to structural and electronic memory, while its extension to magnetic switching in the monolayer limit requires separate evidence.

### 3.4. Competing Magnetic Order and Current-Driven Control in Transition-Metal Chalcogenides

In transition-metal dichalcogenides, structural control can change the magnetic character of the individual layer. Metal coordination, bond lengths, and electronic filling determine the competition among direct exchange, ligand-mediated exchange, and itinerant spin polarization [21,22,24,25]. Strain or substrate coupling can also favor structural reconstruction and charge-density-wave order. This adds a competing electronic and structural response to the stacking-dependent control discussed for chromium trihalides. The crystal phase, stoichiometry, thickness, and interface must therefore be established before different magnetic measurements are compared or assigned to a common ground state.

**Figure 11:**
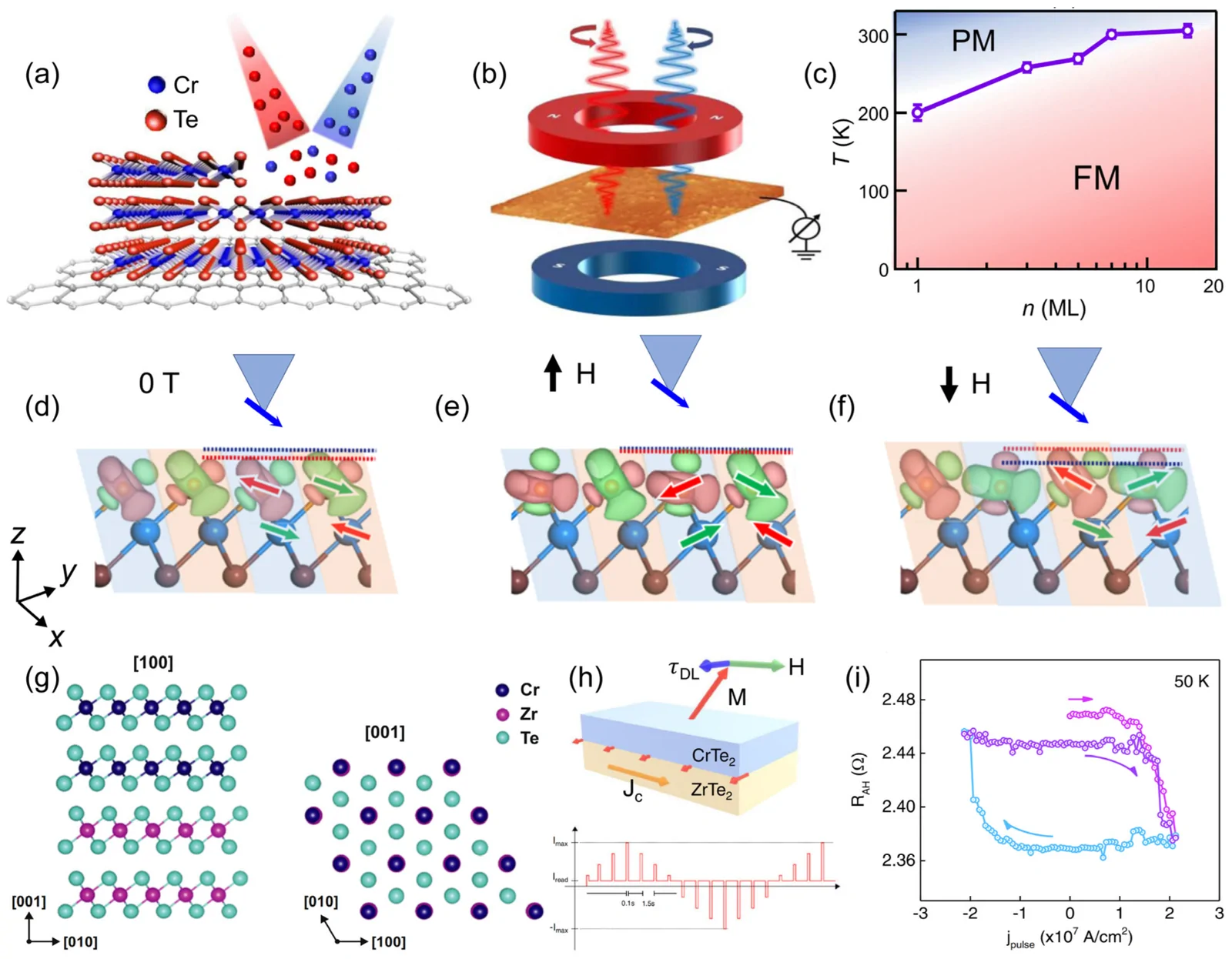
Sample-dependent magnetic order and current-driven control in CrTe_2_. (**a**) Epitaxial-growth schematic, with blue, red, and gray spheres representing Cr, Te, and C atoms, respectively. (**b**) XMCD measurement geometry. Red and blue helical beams denote opposite circular polarizations of the incident X-rays. (**c**) Thickness-dependent magnetic phase diagram showing ferromagnetic (FM) and paramagnetic (PM) regions, shaded red and blue, respectively. From Zhang et al. [60], figures 1a, 3a and 4d. (**d**–**f**) SPSTM measurement models for monolayer CrTe_2_ at zero field, under a positive perpendicular field, and under a negative perpendicular field, respectively. The models illustrate the reorientation of Cr magnetic moments and spin-polarized Te orbitals relative to the tip magnetization, indicated by blue arrows. Red and green arrows show opposing magnetization directions of the Cr moments and Te orbitals. Red and green Te spin-density contours denote spin-up and spin-down polarization, respectively. Black arrows indicate the applied-field direction. Pale red and blue bands distinguish alternating SPSTM contrast. Blue and red dotted lines mark the relative orbital extents toward the tip. From Xian et al. [61], figure 3h–j. (**g**) ZrTe_2_/CrTe_2_ heterostructure viewed along the [100] and [001] directions. Blue, purple, and turquoise spheres represent Cr, Zr, and Te atoms, respectively. (**h**) Spin–orbit torque geometry and current-pulse protocol. Red, green, purple, and orange arrows denote magnetization, assisting magnetic field, damping-like torque, and charge current, respectively. (**i**) Current-induced switching measured through anomalous Hall resistance at 50 K under a 700 Oe assisting field parallel to the current. Purple and cyan arrows indicate opposite current-sweep directions. From Ou et al. [62], figures 1a and 4a,b. Panels (**a**–**c**), (**d**–**f**), and (**g**–**i**) © The Author(s) 2021, 2022, and 2022, respectively, under CC BY 4.0 (https://creativecommons.org/licenses/by/4.0/). Panels cropped, rearranged, and relabeled.

Zhang et al. combined epitaxial growth with element-sensitive magnetic characterization of CrTe_2_ films [60]. The growth and X-ray magnetic circular dichroism (XMCD) geometries in Figure 11a,b identify the preparation route and the Cr-sensitive probe. Their thickness-dependent magnetic phase diagram places the monolayer transition near 200 K, while thicker ultrathin films reach transition temperatures near or above room temperature, as shown in Figure 11c. This result establishes a strong thickness dependence within that sample series. The room-temperature behavior of the thicker films should not be attributed to the monolayer. Thickness, epitaxial environment, and structural characterization remain necessary parts of the material specification.

A different microscopic picture was resolved by Xian et al. in epitaxial monolayer CrTe_2_ [61]. Their spin-polarized scanning tunneling microscopy (SPSTM), interpreted with first-principles calculations, supports zigzag antiferromagnetism. Figure 11d–f illustrates the SPSTM measurement configurations at zero field and under opposite perpendicular magnetic fields. The models show how field-induced reorientation of Cr magnetic moments and the associated spin-polarized Te orbitals changes the tunneling contrast while the tip magnetization remains fixed. These schematics explain the field-dependent contrast observed in the study. The contrast between these antiferromagnetic monolayers and the ferromagnetic sample series above highlights the sensitivity of CrTe_2_ to preparation and its local environment. The comparison does not establish a common ground state for every nominally monolayer CrTe_2_ sample.

Interface engineering can turn an established ferromagnetic film into a current-controlled device. Ou et al. grew ZrTe_2_/CrTe_2_ heterostructures by molecular-beam epitaxy and patterned them for anomalous-Hall readout [62]. Figure 11g shows the heterostructure from two crystallographic directions. The torque geometry and pulse sequence in Figure 11h illustrate how the applied current drives switching and how the magnetic state is read between pulses. The switching loop in Figure 11i was obtained at 50 K in an 8-unit-cell ZrTe_2_/3-unit-cell CrTe_2_ device under a 700 Oe field parallel to the current. The switching onset was approximately 1.8 × 10^7^ A cm^−2^. This provides a low-temperature demonstration of electrical writing in an epitaxial van der Waals stack. The switching loop alone is insufficient to determine a unique torque efficiency.

The same need for sample-specific interpretation extends to other MX_2_ compounds. Reports of monolayer ferromagnetism in VSe_2_ coexist with observations of a nonmagnetic charge-density-wave reconstruction and calculations of competing states [22,24,25]. Strain calculations for CrSe_2_ and CrTe_2_ predict changes in the relative stability of ferromagnetic and antiferromagnetic order, while defects and interface hybridization provide further routes to a magnetic response [21,63,64].

### 3.5. Magnetic Blocks, Carrier Tuning, and Topological Transport in MnBi_2_Te_4_-Family Compounds

The MnBi_2_Te_4_ family links the spacing of magnetic blocks to electronic topology. Inserting nonmagnetic quintuple layers changes the separation between magnetic septuple layers and modifies their coupling [27,28]. Composition and gating additionally change carrier density and the position of the chemical potential [65]. Film thickness controls magnetic compensation and can alter the accessible topological transport regime [9,66]. These variables can therefore influence magnetic and electronic properties through different mechanisms. Establishing a useful control route requires separate evidence for the magnetic configuration, the electronic band state, and the measured transport response.

**Figure 12:**
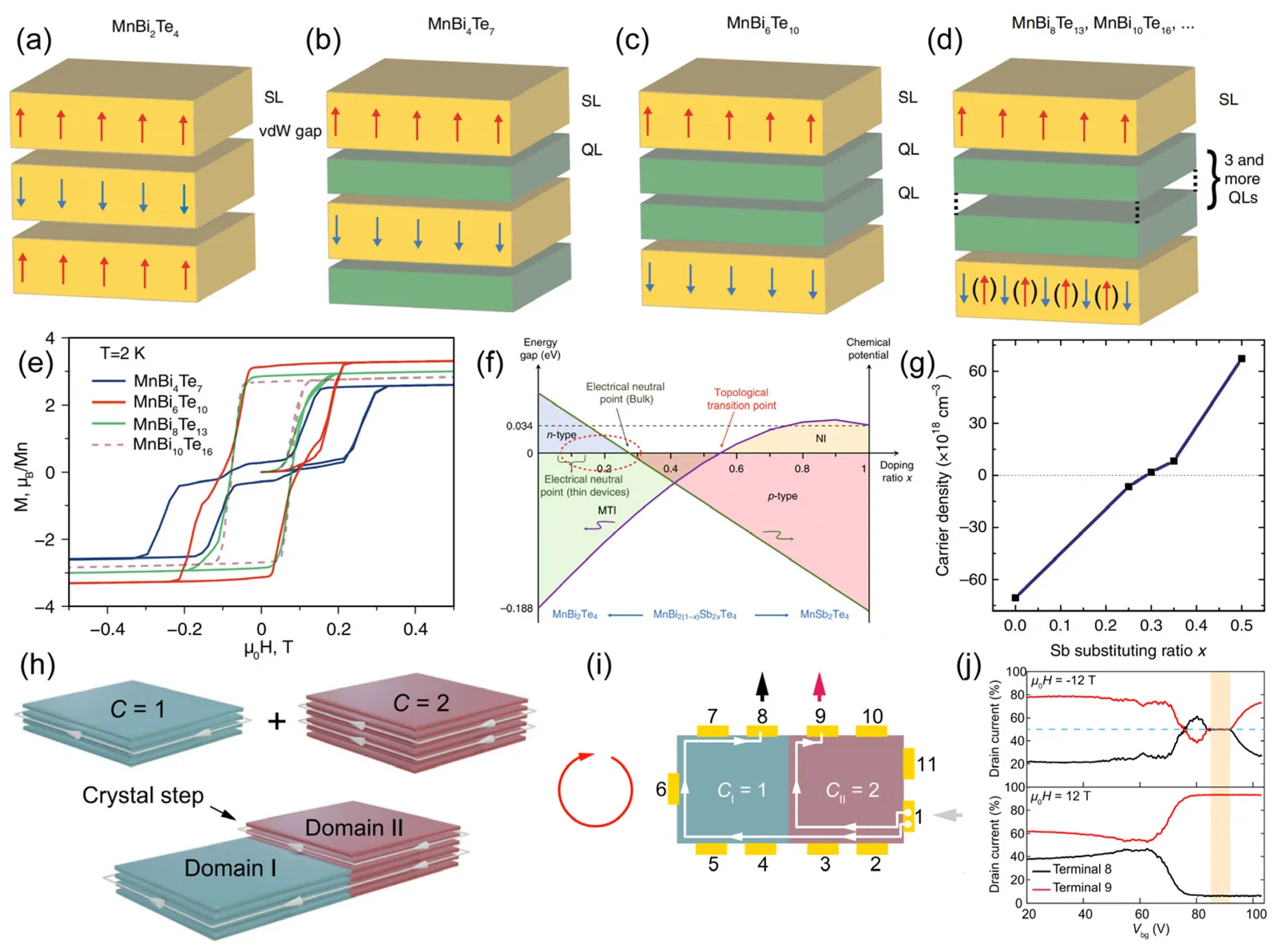
Structural and electronic design in MnBi_2_Te_4_-family materials. (**a**–**d**) Stacking models of MnBi_2_Te_4_, MnBi_4_Te_7_, MnBi_6_Te_10_, and higher members including MnBi_8_Te_13_ and MnBi_10_Te_16_, respectively. The ellipsis in (**d**) denotes higher members of the homologous series. Magnetic septuple layers (SLs) are separated by increasing numbers of nonmagnetic quintuple layers (QLs). Dotted breaks in (**d**) indicate additional QLs omitted from the schematic. (**e**) Field-dependent magnetization of selected homologous compounds at 2 K. The dashed pink curve corresponds to MnBi_10_Te_16_. Panels (**a**–**d**) and (**e**) are from Klimovskikh et al. [28], figures 1a and 1g, respectively. (**f**) Conceptual diagram relating Sb substitution to chemical potential, band gap, and topological regime. The horizontal dashed line marks the 0.034 eV reference. The red dashed ellipse highlights the proposed composition range for realizing the quantum anomalous Hall effect. (**g**) Measured carrier-density evolution with Sb content. The horizontal dashed line marks zero carrier density. From Chen et al. [65], figures 5e and 2i. (**h**) Thickness-step junction connecting MnBi_2_Te_4_ regions with Chern numbers C = 1 and C = 2. (**i**) Edge-channel routing and terminal configuration of the junction. (**j**) Gate-dependent current fractions collected at terminals 8 and 9 at 50 mK under magnetic fields of −12 T (upper panel) and +12 T (lower panel). The blue dashed line marks 50% drain current, corresponding to equal current partition between terminals 8 and 9. From Ovchinnikov et al. [67], figures 1a,b and 2a–c. Panels (**a**–**e**), (**f**,**g**), and (**h**–**j**) © The Author(s) 2020, 2019, and 2022, respectively, under CC BY 4.0 (https://creativecommons.org/licenses/by/4.0/). Panels cropped, rearranged, and relabeled.

The homologous (MnBi_2_Te_4_)(Bi_2_Te_3_)_m_ series separates magnetic septuple layers with nonmagnetic quintuple blocks. Figure 12a–d illustrates the stacking arrangements with zero, one, two, and three or more intervening quintuple layers. Changing the number of these blocks modifies the separation and coupling between magnetic layers. Klimovskikh et al. found different low-temperature magnetic responses across the series, as reflected in the field-dependent magnetization at 2 K in Figure 12e [28]. This comparison motivates control of magnetic dimensionality through the stacking sequence. It does not imply that each added spacer produces an identical or monotonic change in exchange. Magnetic compensation, sample composition, and the balance of weak interactions also matter.

Sb substitution controls the electronic filling within the same layered family. Figure 12f summarizes the proposed relationship among Sb content, chemical potential, and the calculated topological regime. Chen et al. measured a carrier-type change from electron-like to hole-like as the Sb content increased, with compensation near x ≈ 0.3 in their bulk crystals, as shown in Figure 12g [65]. This establishes composition as a route toward reduced bulk carrier density. The conceptual diagram in Figure 12f is not a directly measured topological phase map. The carrier-compensation composition need not coincide with a topological transition or with the value required in a thin device.

Local thickness can translate these material properties into a transport function. Ovchinnikov et al. formed a MnBi_2_Te_4_ junction between regions supporting different Chern numbers [67]. The thickness-step model in Figure 12h connects regions with C = 1 and C = 2. The edge-channel diagram in Figure 12i illustrates how their boundary redistributes current between the drain terminals. Figure 12j compares the measured drain-current fractions at 50 mK under magnetic fields of −12 and +12 T. Gate tuning changes the current partition, while field reversal changes the channel-routing condition. These measurements support a topological current divider in a field-induced Chern-insulator regime; they do not demonstrate zero-field operation of the illustrated device.

Other experiments and predictions broaden the available control routes. Quantum anomalous Hall transport in odd-layer films and field-tuned insulating regimes in even-layer films connect magnetic compensation with topology [9,66]. Surface spin-flop behavior and layer-selective Hall responses show that surface termination and charge distribution can modify the response independently of a complete reversal of the magnetic stack [68,69]. First-principles calculations predict that ferroelectric polarization can switch the topological state of MnBi_2_Te_4_-based heterostructures [70]. Calculations of optically driven lattice modes also predict changes in interlayer magnetic order and band topology in two-septuple-layer antiferromagnets [71]. These proposed responses remain distinct from experimentally demonstrated switching in the corresponding devices.

### 3.6. Spin–Lattice Coupling, Pressure, and Magnetoelectric Readout in Layered Oxyhalides

**Figure 13:**
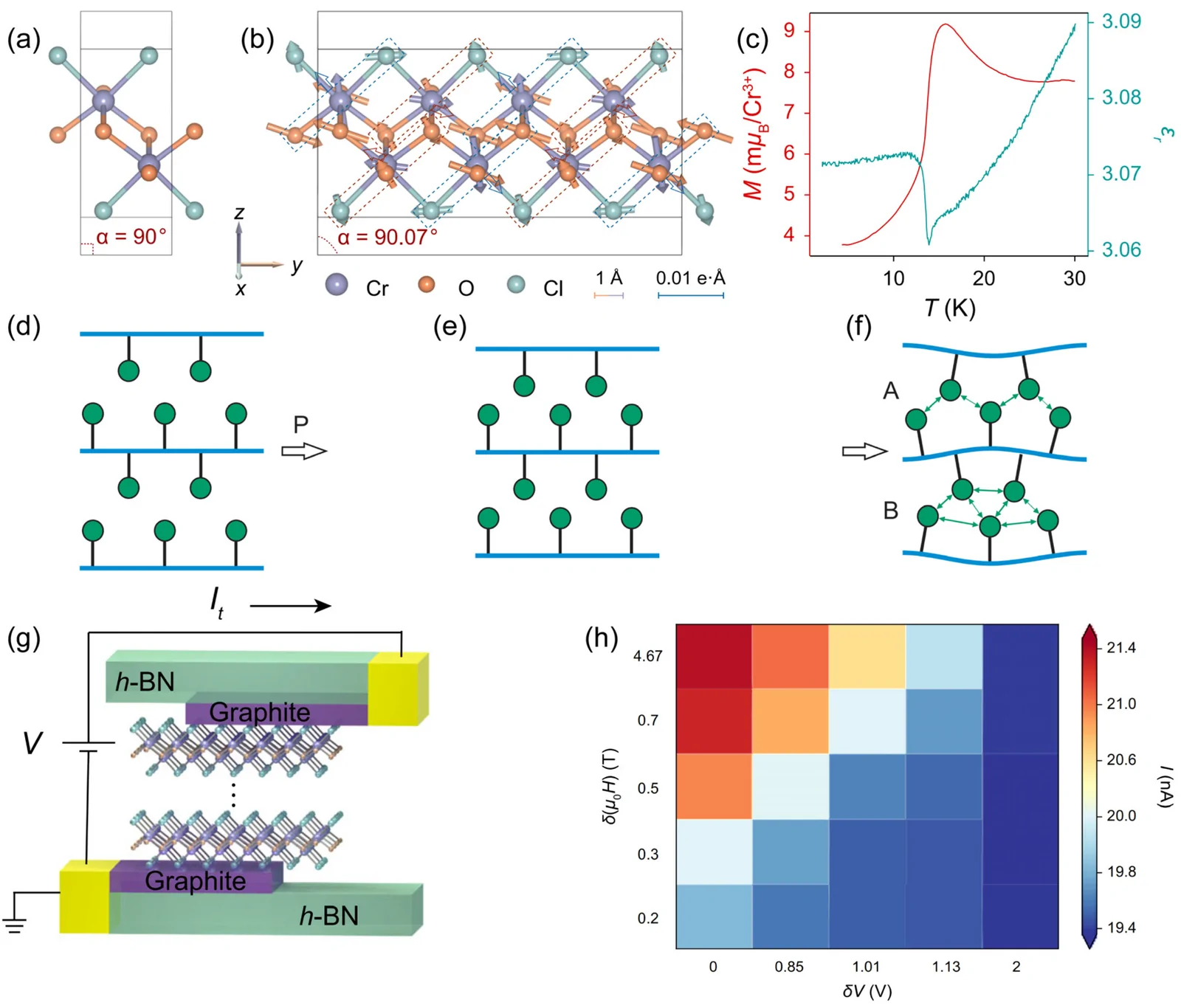
Spin–lattice coupling, pressure-induced structural modulation, and magnetoelectric readout in CrOCl. (**a**,**b**) Structural models above and below the Néel temperature, respectively. The vectors and opposing dipoles in (**b**) represent calculated atomic displacements and antiferroelectric polarization. (**c**) Temperature-dependent magnetic moment and relative permittivity. From Gu et al. [10], figure 1a–c. (**d**–**f**) Schematic evolution from an unmodulated layer arrangement through interlayer compression to a buckled structure. Green circles represent Cl atoms, and double-headed arrows in (**f**) indicate Cl–Cl contacts with more favorable separations after modulation. From Bykov et al. [72], figure 4. (**g**) Graphite/CrOCl/graphite tunneling-device geometry with h-BN encapsulation. (**h**) Tunneling-current matrix after designed electric and magnetic sweeps in an approximately 11.3 nm CrOCl device at 2 K. From Gu et al. [10], figures 2a and 3d. Panels (**a**–**c**,**g**,**h**) and (**d**–**f**) © The Author(s) 2023 and 2015, respectively, under CC BY 4.0 (https://creativecommons.org/licenses/by/4.0/). Panels cropped, rearranged, and relabeled.

The distorted coordination of layered oxyhalides creates directional exchange paths that respond differently to lattice deformation. Magnetic ordering is coupled to changes in bond geometry and crystal symmetry in VOCl, CrOCl, and FeOCl [29,30,31]. This makes pressure and strain effective structural perturbations, while spin–lattice coupling can connect magnetic order to an electrical response. The resulting evidence depends on the measurement. Diffraction establishes structural reorganization, magnetic probes identify spin order, and tunneling detects the coupled device response. Figure 13 illustrates these complementary measurements in CrOCl.

Gu et al. related the stripy antiferromagnetic state of CrOCl to atomic displacements and antiferroelectric polarization [10]. Figure 13a,b compares the structural models above and below the Néel temperature. The displacement vectors and opposing dipoles in Figure 13b represent calculated quantities. The magnetic-moment and relative-permittivity measurements in Figure 13c show anomalies in the same temperature region. Together, these results support a magnetoelectric mechanism linking magnetic order to lattice distortion and local polarization. The structural models illustrate this microscopic coupling but do not directly image individual electric dipoles.

Pressure changes the lattice through a separate, experimentally resolved pathway. Bykov et al. identified an extended incommensurate structural regime in compressed CrOCl and a lock-in transition at higher pressure [72]. Figure 13d–f illustrates the proposed mechanism through an initially unmodulated layer arrangement, interlayer compression, and subsequent buckling. The modulation redistributes Cl–Cl contacts and allows more favorable local separations while accommodating the comparatively rigid Cr–O network. These panels provide a schematic explanation of the structural response. The diffraction results establish pressure-induced structural reorganization and hysteresis; identifying a corresponding magnetic transition requires magnetic measurements under the same conditions.

The coupling can be read electrically in a graphite/CrOCl/graphite tunneling device, as illustrated in Figure 13g. Gu et al. used designed voltage and magnetic-field sweeps to prepare distinguishable current levels [10]. Figure 13h maps the resulting tunneling current against the electric- and magnetic-sweep ranges in an approximately 11.3 nm CrOCl device at 2 K. The retained states reflect the history-dependent coupled system and provide a route to multistate electrical readout. This matrix represents a multilayer device. Although the study also examined monolayer devices, the selected data do not establish the same number of storage states in a monolayer.

The magnetic ground state of monolayer CrOCl requires careful comparison between calculations and experiments. Miao et al. predicted ferromagnetism in an isolated CrOCl monolayer [73]. Qing et al. further calculated strain-induced changes between ferromagnetic and antiferromagnetic states [74]. Their analysis relates this sensitivity to competition between direct Cr–Cr exchange and ligand-mediated superexchange. These findings establish theoretical possibilities for specified lattice structures and strains.

Magnetic and optical studies of bulk and exfoliated CrOCl, together with magnetoelectric tunneling measurements, instead support antiferromagnetic behavior in the investigated samples [10,75]. These experiments do not reproduce all conditions assumed for a free-standing calculated monolayer. A meaningful comparison must therefore specify layer number, lattice relaxation, strain, substrate coupling, and the treatment of electronic correlations. These variables offer possible explanations for the differing conclusions, but the available studies do not isolate a single cause of the discrepancy.

Chen et al. extended sample preparation by growing continuous CrOCl monolayer films and nanosheets through chemical vapor deposition [76]. Their SQUID measurements used stacked nanosheets to obtain sufficient magnetic signal and supported antiferromagnetic ordering near 14 K. The magnetic response of this ensemble should be distinguished from a layer-resolved measurement of a continuous monolayer. Combining controlled growth with thickness-specific magnetic probes would enable a closer comparison with isolated-layer calculations.

Other oxyhalide studies demonstrate control through chemical modification and interfacial charge redistribution. Potassium intercalation changes the composition and magnetic behavior of VOCl [77]. Graphene/CrOCl heterostructures exhibit strong interfacial charge coupling and modified electronic transport [78]. Their transport phase diagrams characterize the coupled heterostructure and do not independently determine a switched CrOCl spin configuration.

The chalcogen halide CrSBr provides a useful comparison with the oxyhalides. Its ferromagnetic layers are coupled antiferromagnetically, which makes their relative alignment sensitive to carrier injection [79,80]. Zhao et al. used gate-controlled lithium intercalation in multilayer devices, including an approximately 6 nm specimen, and obtained magnetotransport evidence for a reversible zero-field antiferromagnetic-to-ferromagnetic transition [79]. Tabataba-Vakili et al. used electrostatic doping in bilayer and trilayer devices to shift magnetic transition fields and probe magnetic states through excitonic spectra [80]. These studies demonstrate distinct electrical control routes. Intercalation changes the chemical state as well as the carrier population, while electrostatic gating tunes charge without intentional ion insertion. Their comparison emphasizes the need to identify the microscopic action of a gate before comparing its magnetic effect across materials.

## 4. Conclusions and Outlook

The comparison of layered van der Waals magnets shows that effective magnetic control depends on identifying the structural or electronic variable that governs the relevant interaction. Layer registry provides access to interlayer exchange, bond deformation modifies exchange geometry, and carrier redistribution changes magnetic stability. Interfaces can introduce additional exchange interactions or supply spin angular momentum. The effectiveness of each route depends on magnetic anisotropy, competing phases, and sample conditions. A shared material label therefore does not guarantee a shared magnetic response.

A first unresolved question is how intrinsic atomic-layer magnetic order can be distinguished from states selected by the surrounding environment. The contrasting results for NiPS_3_, CrTe_2_, VSe_2_, and CrOCl show the importance of thickness, preparation, probe sensitivity, and theoretical assumptions [17,22,24,25,41,60,61,73,74,75]. Measurements that combine structural characterization with complementary magnetic probes on the same specimen are needed. Comparisons should report substrate and encapsulation conditions, composition, and the relevant temperature and field ranges. This would help determine whether different observations reflect distinct magnetic phases or different sensitivities to the same state.

A second question is whether a measured structural change can predict a magnetic response quantitatively. Stacking shifts, bond distortions, and carrier transfer often occur together, which makes their individual contributions difficult to separate. Controlled perturbations should therefore be combined with measurements of local structure, charge distribution, and magnetic excitations. High-throughput calculations can help identify promising structural trends, provided that competing phases, stability, and uncertainty are included [81]. Nonempirical calculations of magnetic excitations offer a complementary route, although methods established for bulk oxides require separate validation in atomically thin magnets [82].

**Figure 14:**
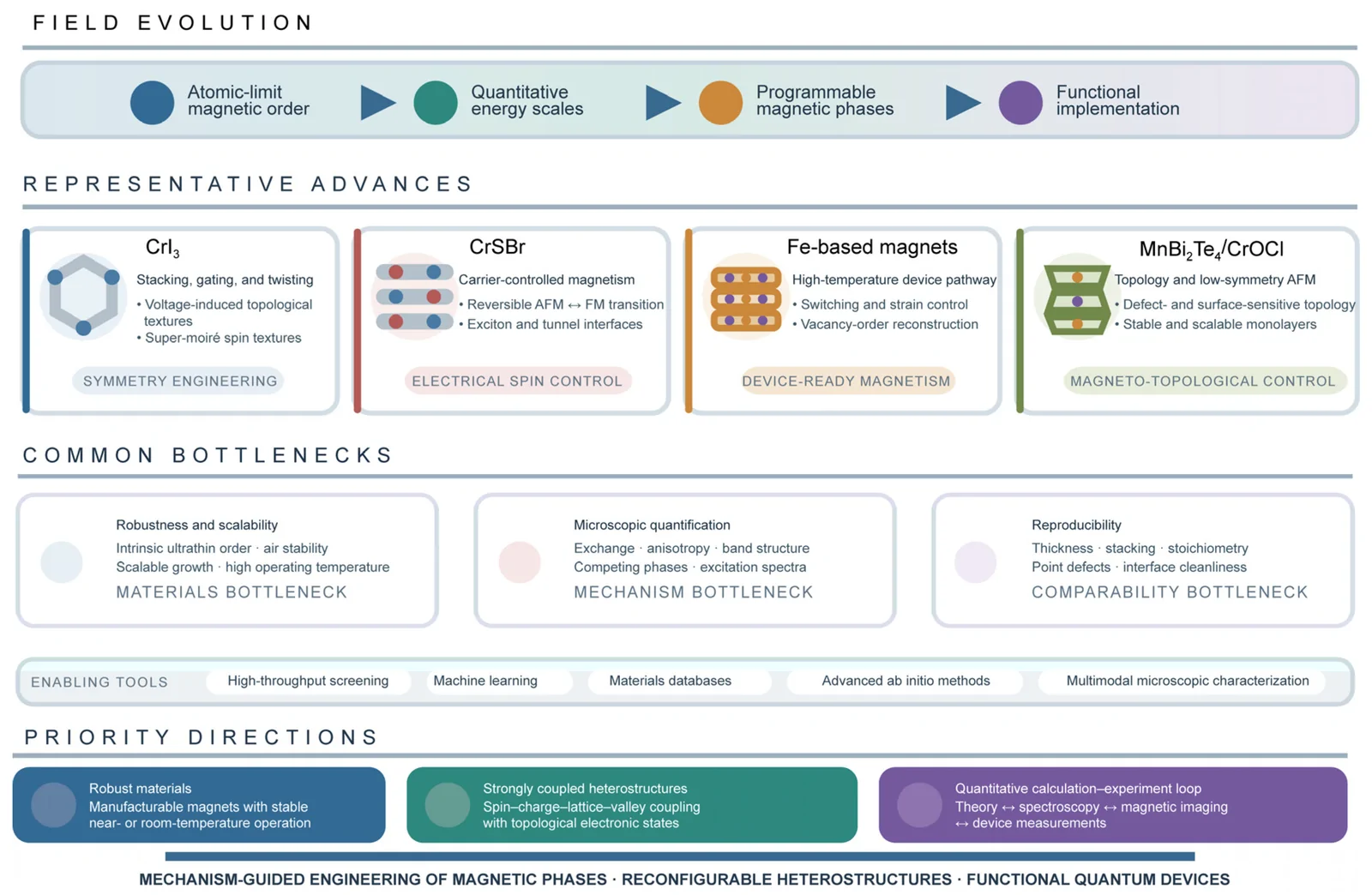
Outlook for controllable two-dimensional magnetism.

A third question concerns the reproducibility and reversibility of controlled magnetic states. The same sensitivity that enables large responses also makes magnetic behavior vulnerable to stacking disorder, defects, and changes in chemical composition. Future experiments should establish whether repeated control cycles preserve the structure and magnetic response across multiple devices. Room-temperature operation, environmental stability, and scalable growth should be tested together under compatible conditions. These shared questions connect the material-specific directions summarized in Figure 14 and define the evidence needed to translate magnetic tunability into reproducible device functions.

## Data Availability

No new data were created or analyzed in this study. Data sharing is not applicable to this article.
